# Additive Promotion of Viral Internal Ribosome Entry Site-Mediated Translation by Far Upstream Element-Binding Protein 1 and an Enterovirus 71-Induced Cleavage Product

**DOI:** 10.1371/journal.ppat.1005959

**Published:** 2016-10-25

**Authors:** Chuan-Tien Hung, Yu-An Kung, Mei-Ling Li, Gary Brewer, Kuo-Ming Lee, Shih-Tung Liu, Shin-Ru Shih

**Affiliations:** 1 Graduate Institute of Biomedical Science, College of Medicine, Chang Gung University, Taoyuan City, Taiwan; 2 Research Center for Emerging Viral Infections, College of Medicine, Chang Gung University, Taoyuan City, Taiwan; 3 Department of Biochemistry and Molecular Biology, Robert Wood Johnson Medical School, Rutgers University, New Jersey, United States Of America; 4 Department of Microbiology and Immunology, College of Medicine, Chang Gung University, Taoyuan City, Taiwan; 5 Clinical Virology Laboratory, Department of Laboratory Medicine, Chang Gung Memorial Hospital, Taoyuan City, Taiwan; Stanford University, UNITED STATES

## Abstract

The 5' untranslated region (5' UTR) of the enterovirus 71 (EV71) RNA genome contains an internal ribosome entry site (IRES) that is indispensable for viral protein translation. Due to the limited coding capacity of their RNA genomes, EV71 and other picornaviruses typically recruit host factors, known as IRES *trans*-acting factors (ITAFs), to mediate IRES-dependent translation. Here, we show that EV71 viral proteinase 2A is capable of cleaving far upstream element-binding protein 1 (FBP1), a positive ITAF that directly binds to the EV71 5' UTR linker region to promote viral IRES-driven translation. The cleavage occurs at the Gly-371 residue of FBP1 during the EV71 infection process, and this generates a functional cleavage product, FBP1^1-371^. Interestingly, the cleavage product acts to promote viral IRES activity. Footprinting analysis and gel mobility shift assay results showed that FBP1^1-371^ similarly binds to the EV71 5' UTR linker region, but at a different site from full-length FBP1; moreover, FBP1 and FBP1^1-371^ were found to act additively to promote IRES-mediated translation and virus yield. Our findings expand the current understanding of virus-host interactions with regard to viral recruitment and modulation of ITAFs, and provide new insights into translational control during viral infection.

## Author Summary

Many RNA viruses utilize internal ribosome entry sites (IRES) located in the 5’ untranslated region of genomic RNA to translate viral proteins in a cap-independent manner. Host proteins that are recruited to assist in viral IRES-driven translation are known as ITAFs (IRES *trans*-acting factors), of which far upstream element-binding protein 1 (FBP1) is an example. In this study, we describe a novel regulatory mechanism involving ITAF cleavage, in which FBP1 is cleaved by EV71 viral proteinase 2A to yield a cleavage product, FBP1^1-371^, which in turn acts additively with full-length FBP1 to enhance viral IRES-mediated translation and virus yield. Footprinting and gel mobility shift analyses reveal that both full-length FBP1 and its cleavage product bind to the linker region of EV71 5′ UTR, but at different sites. To the best of our understanding, these results shed light on a novel interaction between host ITAFs and picornaviruses, and provide important implications for other virus-host interactions.

## Introduction

Enterovirus 71 (EV71), a cytoplasmic RNA virus of the *Picornaviridae* family, is known to infect both animals and humans, and can cause highly fatal neurological complications [1, 2]. During the infection process, EV71 binds to P-selectin glycoprotein ligand-1 (PSGL-1; CD162) [3] or scavenger receptor B2 (SCARB2) [4] on the cell surface, after which the virus enters the cell and releases its genomic RNA into the cytoplasm. The EV71 genome is composed of a 7.4 kb positive-strand RNA, which encodes a single long open reading frame flanked by untranslated regions (UTR). The 5′ terminus of the RNA genome covalently links to a viral protein, VPg, which maintains the stability of the genome and promotes its replication [5, 6]. A poly-adenosine tail at the 3′ terminus of the genome can mimic host mRNA, and also serves to enhance the efficiency of viral replication [7, 8]. The EV71 genome produces a viral polyprotein of about 220 kDa [9, 10], which subsequently undergoes sequence-specific proteolytic cleavage by the viral proteinases 2A (2A^pro^) and 3C (3C^pro^) [11], as well as other *cis* and *trans* modifications, which eventually yield mature viral proteins and precursor molecules that act to foster a suitable environment for viral propagation.

Due to the absence of a 7-methyl guanosine (m7G) cap, the EV71 genome cannot be recognized by the host eIF4F cap-binding complex to undergo cap-dependent translation. The 5′ UTR of EV71 contains six RNA stem-loop structures, with stem-loop I (also known as the “cloverleaf”) acting as a viral replication element at the 5′ end [12, 13], and stem-loops II to VI forming an internal ribosome entry site (IRES) that facilitates ribosome recruitment for IRES-dependent translation [13, 14]. IRES-mediated translation primarily depends on canonical cellular translation factors, as well as auxiliary factors known as IRES *trans*-acting factors (ITAFs). The coding limitations of their small viral genomes have led picornaviruses to evolve mechanisms for the exploitation of host factors to facilitate viral protein translation and genome replication, and therefore many host proteins are recruited as ITAFs that can interact with the viral IRES and modulate IRES-driven translation. For example, members of the heterogeneous ribonucleoprotein (hnRNP) family such as polypyrimidine tract-binding protein (PTB) [15, 16], poly(rC)-binding protein 2 (PCBP2) [17–19], AU-rich element binding factor 1 (AUF1) [20], and the cellular nuclear proteins, far upstream element-binding protein 1 (FBP1) and 2 (FBP2) [21, 22], have all been found to act as ITAFs that regulate IRES activity via direct binding to distinct regions of the 5′ UTR [23, 24].

Besides the recruitment of host proteins to assist with viral protein expression and replication, picornaviruses have also been found to modify host factors through viral enzymatic cleavage to facilitate viral propagation. For example, viral 2A^pro^ can cleave eukaryotic initiation factor 4G (eIF4G) [25, 26] and poly(A)-binding protein (PABP) [27, 28], and this subsequently disables the cap-dependent translational complex responsible for initiating translation of host mRNA. Viral proteinase 3C/3CD is known to cleave host cleavage stimulation factor subunit 64 (CstF-64), leading to severe inhibition of cellular mRNA polyadenylation [29]; in addition, the cellular ITAFs, PTB and PCBP2, are also cleaved by viral proteinase 3C/3CD [30, 31]. PTB and PCBP2 are indispensable for the stimulation and stabilization of IRES-driven translation, and cleavage of these two host proteins can trigger switches in viral genome template usage between viral translation and viral replication [30, 31].

FBP1 is a 644-amino acid cellular nuclear protein that comprises three major domains: an amphipathic helix domain in the N-terminus, four hnRNP K homologous domains (KH domain) in the central region, and a C-terminal tyrosine-rich transactivation domain [32]. The KH domains of FBP1 possess DNA and RNA binding ability, and participate in multiple cellular processes, including gene transcription, mRNA degradation, and modulation of mRNA translation. FBP1 has been reported to bind to the far upstream element (FUSE) located in the upstream region of the *c-myc* promoter, acting to promote maximum transcriptional activity of *c-myc* via its C-terminal tyrosine-rich transactivation domain [33–35]. FBP1 is also known to modulate mRNA stability by binding to the 3′ UTR of growth-associated protein 43 (GAP43) mRNA to promote its degradation [36]. FBP1 is further known to bind to the 5′ UTR of p27Kip1 mRNA to enhance translation, but can also bind with the 3′ UTR of nucleophosmin to negatively regulate its translation [37, 38]. In addition to these cellular roles, FBP1 is often recruited by viruses to enhance viral propagation. For example, FBP1 can interact with the poly(U/UC) tract region within the 3′ UTR of the hepatitis C virus (HCV) to promote efficient viral replication [39]. FBP1 has also been found to bind with the 3′ UTR of Japanese encephalitis virus (JEV) RNA to act as a negative regulator that suppresses protein translation and viral replication [40].

In a previous study, we showed that FBP1 directly interacts with the linker region in the 5′ UTR of EV71 to serve as a positive regulator that enhances viral translation and viral growth [21]. In this study, we describe an intriguing viral-induced modification of FBP1 that occurs during EV71 infection. An *in vitro* cleavage assay using recombinant viral proteinases and isotopic-labeled substrates revealed that FBP1 is cleaved at glycine residue 371 by EV71 viral 2A^pro^. Mutant FBP1^G371K^ overexpressed in EV71-infected RD cells was resistant to viral 2A^pro^ cleavage, and this provides further evidence that glycine residue 371 is the authentic cleavage site of viral 2A^pro^. Both full-length FBP1 and truncated FBP^1-371^ can bind to the linker region of the EV71 genome, and we further found that truncated FBP^1-371^ acts additively with full-length FBP1 to enhance viral IRES activity and virus yield. Taken together, these results present a novel mechanism of viral-induced ITAF modulation in EV71 infection.

## Results

### Cleavage of FBP1 during EV71 infection

**Fig 1:**
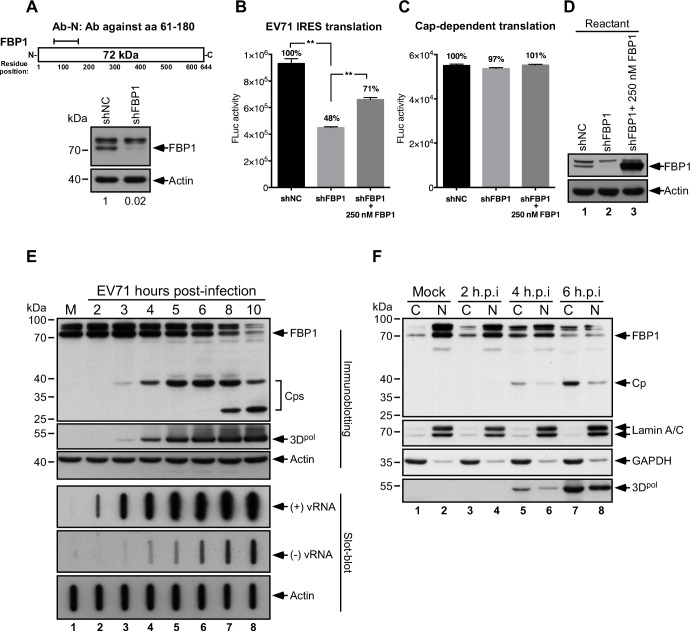
Function, expression and subcellular localization of FBP1 in EV71-infected cells. (A) RD cells were transduced with lentivirus carrying shNC (control) or shFBP1. Knockdown efficiency of FBP1 was confirmed by immunoblotting with antibodies that recognize the N-terminal epitope (Ab-N) of FBP1. Signal intensities of immunoblotting were quantified by ImageJ software, and the ratios of FBP1 intensity to actin are shown at bottom. *In vitro* (B) IRES-dependent and (C) cap-dependent translation were performed using EV71 5′ UTR-FLuc monocistronic RNA. Reactions were incubated with shNC or shFBP1 RD cytoplasmic extracts in the presence or absence of 250 nM of recombinant FBP1, and (D) 50% of the translation reactant were subjected to immunoblotting. Luciferase activity exhibited by the reporter was monitored with a luminometer. Error bars represent the standard deviation from three experimental and three technical replicates. P value was determined by two-tailed Student’s t test (**, *p* < 0.01). (E) Proteins expressed by RD cells at 2–10 hours post-infection (h.p.i.) by EV71 were analyzed by immunoblot analysis using anti-FBP1, anti-3D^pol^, and anti-actin antibodies. Cp: Cleavage product of FBP1. Viral RNA was extracted at 2–10 h.p.i. and analyzed by slot-blotting. (F) Proteins in the cytoplasmic and nuclear fractions taken from cells at 2–6 h.p.i. were analyzed by immunoblot analysis, using anti-FBP1, anti-Lamin A/C, anti-GAPDH, and anti-3D^pol^ antibodies. M: mock-infected cells; C: cytoplasmic fraction; N: nuclear fraction.

To elucidate how FBP1 regulates EV71 IRES-driven translation, we used RD cells that were transduced with lentivirus expressing FBP1 shRNA. Immunoblot analysis using antibodies that recognize the N-terminal regions of FBP1 (Ab-N, Fig 1A, top panel) revealed that the shRNA reduced FBP1 expression in RD cells (Fig 1A), and an *in vitro* translation assay using EV71 5′ UTR-FLuc reporter RNA and shFBP1-RD cytoplasmic extracts revealed that FBP1 shRNA reduced EV71 IRES-driven translation by 52% (Fig 1B). The addition of 250 nM of FBP1, which was purified from SF9 cells expressing FBP1, partially restored activity, with an observed reduction in IRES-driven translation of only 29% (Fig 1B). However, FBP1 shRNA expression or the addition of purified FBP1 did not affect Cap-Luc RNA translation (cap-dependent translation) (Fig 1C). Levels of FBP1 in the reactions were also confirmed by immunoblotting (Fig 1D). These results indicate that FBP1 promotes IRES-driven translation, but does not affect cap-dependent translation.

This study also examined FBP1 expression in RD cells following EV71 infection at a m.o.i. of 40. Decreased FBP1 expression was evident at 4 hours post-infection (h.p.i.), and FBP1 levels were significantly reduced by 8 and 10 hours post-infection (Fig 1E). In addition, a 38-kDa protein band, likely a cleavage product (Cp) of FBP1, appeared at Hour 4 and reached a maximal level at Hour 6; moreover, a potential 30-kDa Cp of FBP1 appeared at 8 and 10 hours post-infection, demonstrating that EV71 infection destabilizes FBP1 (Fig 1E). Total RNA from infected cells was further examined by slot blot analysis. After UV-crosslinking to membranes, viral RNA was hybridized with DIG-labeled RNA probes, specific for the detection of sense (+) or anti-sense (−) viral RNA (vRNA). Following hybridization, blots were washed and incubated with chemiluminescent substrate for visualization. Results showed that levels of both (−) vRNA and (+) vRNA increased over a 10-hour period following EV71 infection (Fig 1E). Meanwhile, nuclear and cytoplasmic fractions were collected and subjected to immunoblot analysis, and the results indicated that FBP1 remained primarily in the nucleus during mock infection (Fig 1F, lanes 1 and 2), but appeared in the cytoplasmic faction at increasing levels over 2 to 6 h.p.i. (Fig 1F, lanes 3, 5, and 7). In contrast to FBP1, Cp was mostly present in the cytoplasm (Fig 1F, lanes 5 and 7), and Cp levels increased through the course of infection. Taken together, these results demonstrate that FBP1 promotes EV71 IRES-mediated translation, and the FBP1 protein is likely subject to proteolytic cleavage during the middle stage of EV71 infection.

### FBP1 cleavage is independent of proteasome, lysosome, and caspase activities

**Fig 2:**
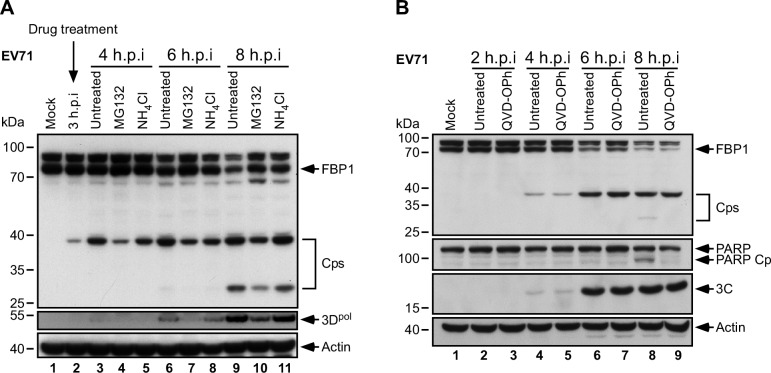
FBP1 cleavage following EV71 infection is independent of cellular proteasome, lysosome, or caspase activities. (A) EV71-infected or mock-infected RD cells were treated with 20 μM MG132 or 20 mM NH_4_Cl at 3 h.p.i. Cell extracts at the indicated time points were analyzed with anti-FBP1, anti-3D^pol^ and anti-actin antibodies. Viral 3D^pol^ was used as an indicator for viral replication. (B) Cells were treated with 20 μM QVD-OPh, and lysates from the indicated time points were analysed with anti-FBP1, anti-PARP, anti-3C and anti-actin antibodies. Detection of PARP and its cleavage product (PARP Cp) was used as a positive control for viral-induced caspase activity. Viral 3C protein was used as an indicator for virus infection, and actin served as a loading control. Cps: Cleavage products of FBP1.

An earlier study showed that far upstream element-binding protein 2 (FBP2), a nuclear-resident protein of the same family as FBP1, was cleaved by proteasomes and lysosomes following EV71 infection [41]. We therefore investigated whether FBP1 was similarly cleaved after EV71 infection. RD cells infected with EV71 were respectively treated with either proteasome inhibitor MG132 or lysosome inhibitor NH_4_Cl at 3 h.p.i, and subsequently subjected to immunoblot analysis using Ab-N against FBP1. Immunoblotting results of cell lysates revealed that the treatments did not decrease levels of FBP1 cleavage (Fig 2A). As FBP1 is known to be a substrate for caspase-3 and caspase-7 [42], and EV71 infection induces caspase activation [43, 44], we treated EV71-infected RD cells with a pan-caspase inhibitor, QVD-OPh. Results showed that PARP, a known substrate of caspase-3, was cleaved in EV71-infected cells (Fig 2B, lane 8), and the cleavage of PARP was inhibited with QVD-OPh treatment (Fig 2B, lane 9); however, even with the addition of QVD-OPh, FBP1 levels continued to decline after 4 h.p.i., and the 38-kDa Cp was detectable at 4–8 h.p.i. (Fig 2B), indicating that caspase inhibition was unable to prevent FBP1 cleavage. Incidentally, the 30-kDa Cp became undetectable following QVD-OPh treatment (Fig 2B), suggesting that this band represents the caspase cleavage product of FBP1. Together, these results show the primary Cp of FBP1 following EV71 infection is not derived from proteasome, lysosome, or caspase activities.

### Cleavage of FBP1 by EV71 viral proteinase 2A *in vitro*

**Fig 3:**
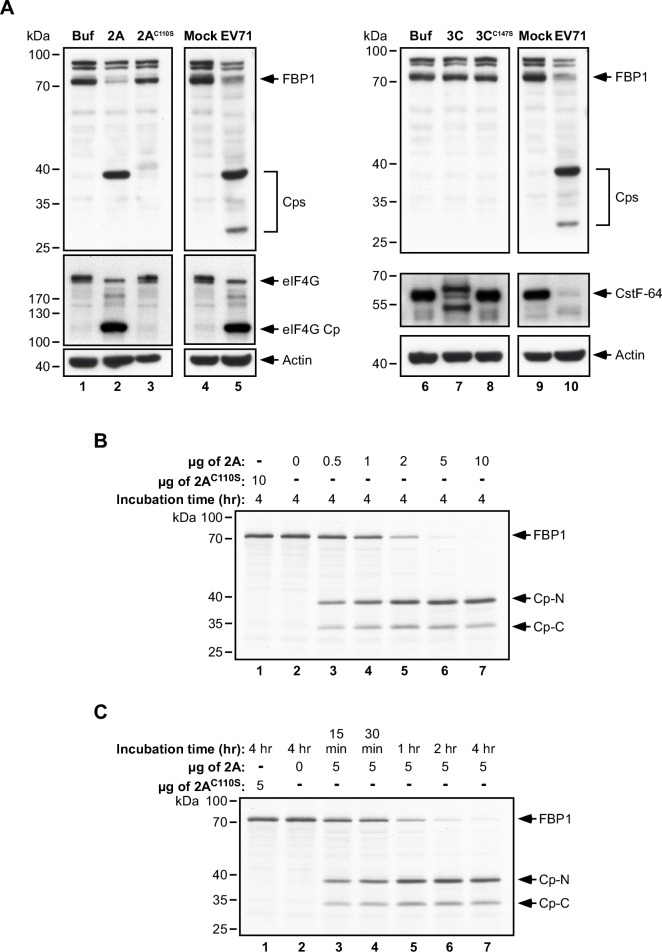
*In vitro* induction of FBP1 cleavage by EV71 viral proteinase 2A. (A) 10 μg of wild-type 2A^pro^ (2A) or mutant 2A^pro^ (2A^C110S^), wild-type 3C^pro^ (3C) or mutant 3C^pro^ (3C^C147S^) viral proteinases were added to RD cell lysates and incubated for 4 hours at 37°C. Cleavage of eIF4G and CstF-64 respectively served as positive controls for 2A^pro^ and 3C^pro^ activity. (B) [^35^S] methionine-labeled FBP1 was incubated with purified EV71 2A^pro^ or mutant 2A^pro^ (2A^C110S^) for 4 hours at varying doses, or for (C)15 minutes to 4 hours at a fixed dose of 5 μg at 37°C. Proteins were then separated by SDS-PAGE and analyzed by autoradiography. Cp-N and Cp-C: Cleavage products of FBP1.

As the cleavage of FBP1 was shown to be independent of host cellular pathways (Fig 2), we therefore sought to determine whether FBP1 cleavage was caused by viral factors. We respectively added 10 μg of purified recombinant EV71 viral wild-type 2A^pro^ (2A), catalytic defective mutant 2A^pro^ (2A^C110S^), wild-type 3C^pro^ (3C), or catalytic defective mutant 3C^pro^ (3C^C147S^) to lysates prepared from RD cells. Following incubation at 37°C for 4 hours, proteins in the lysate were analyzed by immunoblotting using Ab-N of FBP1. In control experiments, we confirmed that wild-type 2A^pro^ (2A) was able to cleave its substrate, eIF4G, as expected (Fig 3A, lane 2), while mutant 2A^C110S^ did not cleave eIF4G (Fig 3A, lane 3). Immunoblotting results further revealed that wild-type 2A^pro^ cleaved FBP1 (Fig 3A, lane 2) to produce a 38-kDa cleavage product (Cp), which migrated to the same position in a gel as the Cp band observed in EV71-infected cells (Fig 3A, lane 5, Cps upper band). However, FBP1 remained uncleaved if 2A^pro^ was not added to the lysate (Fig 3A, lane 1), or if mutant 2A^pro^ without proteolytic activity was added (Fig 3A, lane 3). A parallel experiment was performed to ascertain whether FBP1 could be cleaved by another EV71-encoded proteinase, 3C^pro^, and the results showed that wild-type 3C^pro^ cleaved a known substrate, CstF-64, but did not cleave FBP1 (Fig 3A, lane 7). We also noticed a slower migrating band beyond the position of CstF-64 after 3C^pro^ treatment; however, this band was undetectable when we applied another CstF-64 antibody (S1 Fig), indicating that this may be a cross-reaction of the originally used CstF-64 antibody with an unknown cellular 3C^pro^ substrate. In order to clarify cleavage patterns and detect any additional cleavage products that might be overlooked due to lack of epitope reactivity with the anti-FBP1 antibody used, we generated [^35^S] methionine-labeled FBP1 via *in vitro* transcription and translation, and autoradiography revealed that FBP1 was cleaved by 2A^pro^ in a dose-dependent manner that resulted in two major Cps (Fig 3B, lanes 3–7), a 38-kDa Cp designated as Cp-N, which was consistent in size with the Cp band detected in RD cells following EV71 infection, and a 33-kDa Cp that was designated as Cp-C. We further examined the cleavage kinetics of FBP1 by incubating 5 μg of 2A^pro^ with [^35^S] methionine-labeled FBP1, and assayed the reactions at various time points. Cp-N and Cp-C were detected after 15 minutes of incubation (Fig 3C, lane 3), and levels of Cp-N and Cp-C increased over the 4 hours of incubation observed (Fig 3C, lanes 3–7). By Hour 4, [^35^S] methionine-labeled FBP1 was almost undetectable, with only Cp-N and Cp-C observed (Fig 3C, lane 7). These results provide evidence that the EV71 viral 2A^pro^ is capable of cleaving FBP1.

### Mapping the EV71 2A^pro^ cleavage site in FBP1

**Fig 4:**
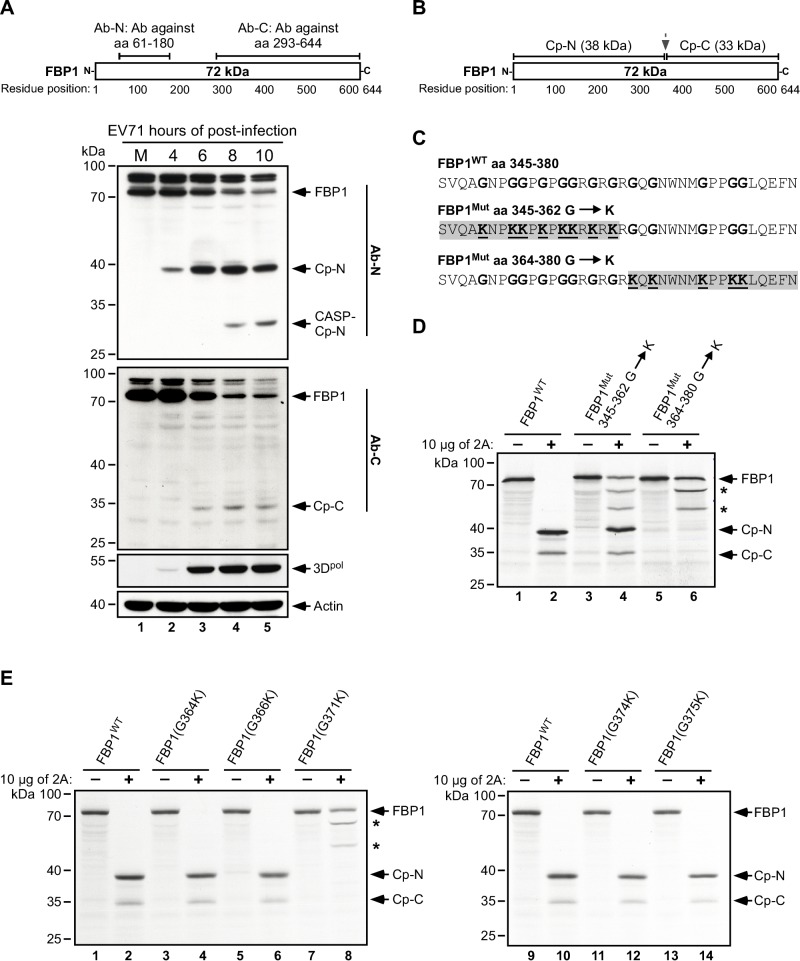
Determining the EV71 2A^pro^ primary cleavage site in FBP1. (A) Antibodies that recognize the N-terminal epitope (Ab-N) or C-terminal epitope (Ab-C) of FBP1 were used to detect FBP1 and its cleavage products (Cp-N and Cp-C) in mock-infected or EV71-infected RD cell lysates prepared at 4, 6, 8, and 10 h.p.i. Viral 3D protein was used as an indicator for virus infection, and actin served as a loading control. Cp-N and Cp-C: Cleavage products respectively including the N-terminal or C-terminal region of FBP1; CASP-Cp-N: a caspase-induced cleavage product of Cp-N. (B) Schematic representation of the predicted cutting sites in FBP1 by 2A^pro^; and (C) potential 2A^pro^ glycine cleavage sites (boldface type) between aa 345 to 380 in FBP1. The regions which contain glycine to lysine mutations in FBP1 are highlighted in gray. (D) [^35^S] methionine-labeled FBP1 (WT) and mutant FBP1 (Mut) were respectively incubated with purified EV71 2A^pro^ (2A) in cleavage buffer for 4 hours at 37°C. Proteins in the lysates were separated by SDS-PAGE and analyzed by autoradiography. Non-specific cleavage products are indicated with an asterisk. (E) [^35^S] methionine-labeled FBP1(G364K), FBP1(G366K), FBP1(G371K), FBP1(G374K) and FBP1(G375K) were treated with EV71 2A^pro^ (2A), and proteins were analyzed by autoradiography after SDS-PAGE.

To determine the 2A^pro^ cleavage site in FBP1, we first analyzed patterns of cleaved FBP1 in EV71-infected RD cell lysates by immunoblot analysis, using antibodies that respectively recognize the N-terminal (Ab-N) and C-terminal (Ab-C) regions of FBP1 (Fig 4A, top panel). These two antibodies respectively detected a 38-kDa (Cp-N) and a 33-kDa (Cp-C) FBP1 cleavage product (Fig 4A, lower panel, lanes 3–5). Previous studies on picornaviruses have revealed that 2A^pro^ preferentially cuts at glycine residues [45, 46], and therefore, based on the number and size of the cleavage products observed, we predicted that 2A^pro^ likely cuts at a glycine residue located between aa 345–380 of FBP1 (Fig 4B and 4C). To determine the exact site at which 2A^pro^ cleaves FBP1, we introduced mutations that substituted glycine residues with lysine in the target regions on FBP1, which should technically prevent cleavage by 2A^pro^ [47] (Fig 4C). Cleavage of [^35^S] methionine-labeled FBP1 *in vitro* by 2A^pro^ revealed that FBP1 mutants with all glycine residues mutated in the region from aa 345 to 362 were cut by 2A^pro^ (Fig 4D, lane 4), indicating that the protease cleavage site is not located within this region. However, mutation of all glycine residues located between aa 364 and 380 prevented cleavage (Fig 4D, lane 6), confirming that the cleavage site is located within this region. Notably, two additional non-specific cleavage products of FBP1 were detected after mutating the cleavage site (Fig 4D, lane 4 and 6, indicated by asterisks). As these additional cleavage products were not observed in the 2A^pro^ cleavage profile of FBP1 (Fig 3C), this suggests that they do not represent 2A^pro^ cleavage products or cleavage intermediates. The non-specific cleavage of FBP1 may be due to blockage of the primary cleavage site through glycine-to-lysine mutation, perhaps forcing 2A^pro^ to cleave at alternative locations of FBP1, or causing conformational changes in FBP1 that leads to the exposure of alternative non-favored cleavage sites for 2A^pro^. In order to pinpoint the primary cleavage site, single glycine-to-lysine mutations were introduced to generate G364K, G366K, G371K, G374K, and G375K substitutions. Our results showed that of all the mutants tested, FBP1^G371K^ alone could not be cleaved by 2A^pro^, and Cp-N and Cp-C cleavage products were not observed (Fig 4E), thereby providing *in vitro* evidence that the Gly-371 residue of FBP1 is likely the primary cleavage site for EV71 viral proteinase 2A.

**Fig 5:**
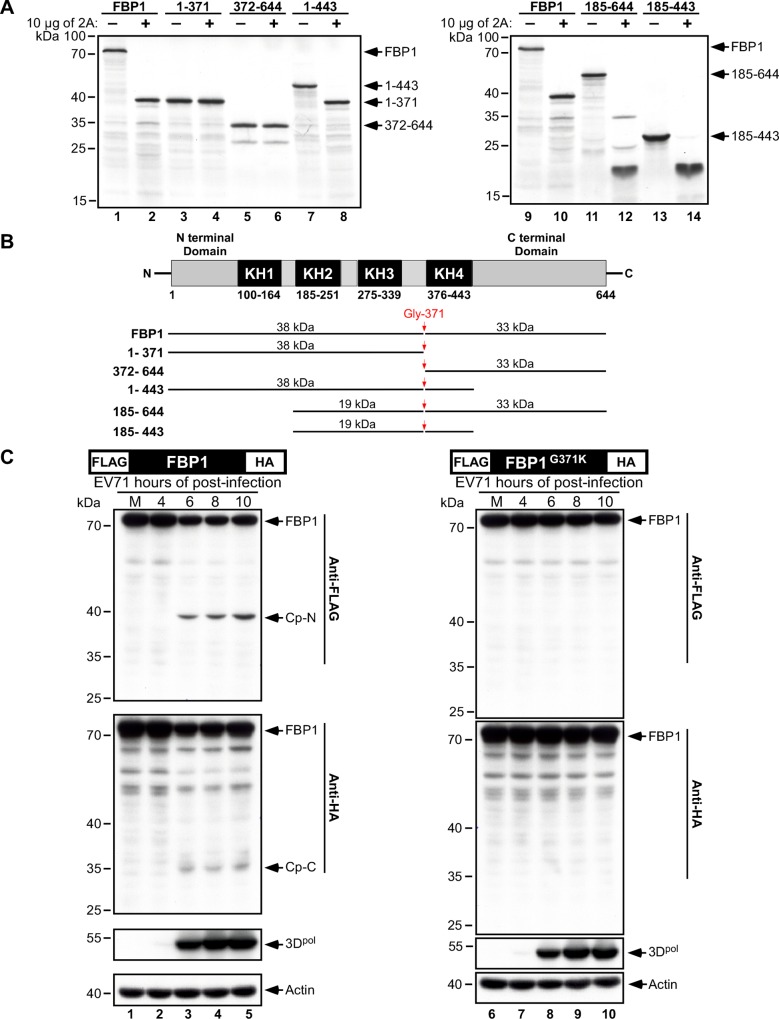
Confirmation of FBP1 cleavage at Gly-371 by EV71 2A^pro^. (A) [^35^S] methionine-labeled FBP1 and FBP1 fragments containing aa 1–371, 372–644, 1–443, 185–644, and 185–443 were treated (+) or untreated (-) with EV71 2A^pro^ (2A). (B) Schematic representation of FBP1, FBP1 fragments and the proposed primary cleavage site at Gly-371 (indicated by an arrow). The molecular masses of the corresponding cleavage products are also shown. (C) RD cells transfected with FLAG-HA dual-tagged FBP1 and mutant FBP1^G371K^, the latter of which is resistant to 2A^pro^ cleavage, were infected with EV71. At 4, 6, 8 and 10 h.p.i., cell lysates were prepared and analyzed by immunoblotting with anti-FLAG, anti-HA, anti-EV71 3D^pol^ and anti-actin antibodies. Cleavage products containing the FLAG-tag or HA-tag are indicated as Cp. Cp-N and Cp-C: Cleavage products respectively containing the N-terminal or C-terminal region of FBP1.

To ascertain whether FBP1 is only cleaved by EV71 viral 2A^pro^ at Gly-371, [^35^S] methionine-labeled full-length FBP1 and fragments containing the sequences from aa 1–371, 372–644, 1–443, 185–644, and 185–443 were incubated with 2A^pro^ *in vitro* (Fig 5A). Fig 5B presents the sizes of these fragments, as well as their predicted cleavage products in the case of a single cleavage site at Gly-371. The results showed that FBP1 fragments 1–371 and 372–644 were not cut by 2A^pro^, and the sizes of these fragments were consistent with that of Cp-N and Cp-C (Fig 5A). In contrast, fragments containing the Gly-371 cleavage site, i.e. 1–443, 185–644, and 185–443 were cleaved after 2A^pro^ treatment. Fragment 1–443 yielded an observable 38-kDa product (Fig 5A, lane 8), whereas fragments 185–644 and 185–443 generated similar 19-kDa cleavage products (Fig 5A, lanes 12 and 14); in addition, a 33-kDa product was also seen with fragment 185–644 (Fig 5A, lanes 12). The patterns of the cleavage products seen in Fig 5A indicate that 2A^pro^ likely cuts only at the Gly-371 residue of FBP1, and to further confirm that this occurs during EV71 infection, we expressed FLAG-HA dual-tagged FBP1 and FBP1^G371K^ in RD cells, followed by EV71 infection (Fig 5C). Immunoblot analysis revealed that during the course of infection, FLAG-FBP1-HA was cleaved, and Cp-N and Cp-C were respectively detected by anti-FLAG and anti-HA antibodies (Fig 5C, lanes 3–5). However, FLAG-FBP1^G371K^-HA was resistant to 2A^pro^ cleavage *in vivo*. Taken together, these results confirm that FBP1 is cleaved at Gly-371 by viral 2A^pro^ during the course of EV71 infection, and the Gly-371 residue is likely the primary cleavage site.

### Association of FBP1, FBP1^1-371^ and EV71 5′ UTR RNA

**Fig 6:**
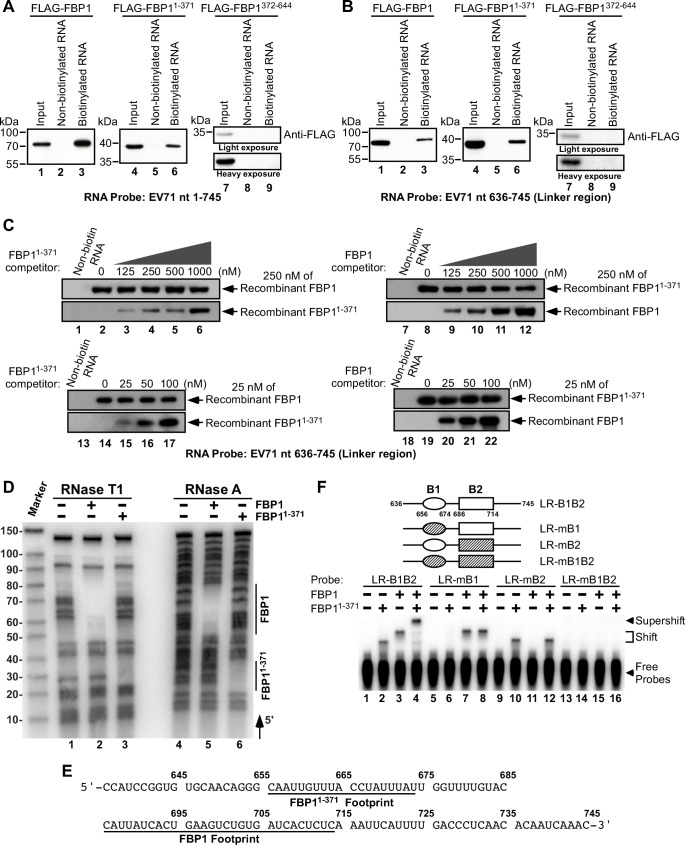
Association between FBP1, FBP1^1-371^, and the EV71 5′ UTR. (A) An EV71 5′ UTR RNA probe (EV71 nt 1–745) either labeled (lanes 3, 6, 9) or unlabeled (lanes 2, 5, 8) with biotin was added to lysates from RD cells that had been respectively transfected with plasmids expressing FLAG-tagged FBP1, FBP1^1-371^ or FBP1^372-644^. Streptavidin beads were then added to the mixture, and proteins pulled down by the beads were analyzed by immunoblotting with anti-FLAG antibody. Input lanes were loaded with 15% of the cell extracts. (B) An EV71 5′ UTR linker region RNA probe (EV71 nt 636–745), either labeled (lanes 3, 6, 9) or unlabeled (lanes 2, 5, 8) with biotin, was added to lysates from RD cells that had been transfected with plasmids expressing either FBP1, FBP1^1-371^ or FBP1^372-644^. Proteins bound to the probes were analyzed by immunoblotting with anti-FLAG antibody. Input lanes were loaded with 15% of the lysates. (C) *In vitro* competition assay of FBP1^1-371^ and FBP1 binding to EV71 5′ UTR linker region RNA. Designated amounts of recombinant FBP1^1-371^ and FBP1 were incubated with EV71 5′ UTR linker region RNA probe, and analyzed by immunoblotting with anti-His antibody. (D) Enzymatic RNA footprinting of FBP1 and FBP1^1-371^ bound to EV71 5′ UTR linker region RNA. Cleavage products of ^32^P-labeled EV71 5′ UTR linker region RNA digested by RNase T1 and RNase A, with or without FBP1 and FBP1^1-371^, were analyzed by denaturing acrylamide gel electrophoresis. Vertical lines on the right of the gel indicate nucleotides that are protected from RNase digestion in the presence of FBP1 and FBP1^1-371^, and (E) detailed binding sites of FBP1 and FBP1^1-371^ within the EV71 5′ UTR linker region RNA are illustrated. (F) Gel mobility shift assays of the FBP1 and FBP1^1-371^ binding sites within the EV71 5′ UTR linker region. The radiolabeled linker region probe used in lanes 1–4 (LR-B1B2) correspond to the linker region sequence (EV71 nt 636–745) that contains the FBP1^1-371^ binding site (B1: EV71 nt 656–674) and FBP1 binding site (B2: EV71 nt 686–714). The LR-mB1 probe used in lanes 5–8 contains FBP1^1-371^ binding site mutations, while the LR-mB2 probe used in lanes 9–12 contains FBP1 binding site mutations. The LR-mB1B2 probe used in lanes 13–16 contained mutations at both FBP1^1-371^ and FBP1 binding sites. The designated probes were respectively incubated with recombinant FBP1^1-371^ alone, FBP1 alone, or both, and shifts and supershifts generated by the formation of protein-RNA complexes are indicated.

To address the roles of cleaved FBP1 proteins in EV71 IRES activity, we first tested the binding capabilities of the cleaved FBP1 proteins, FBP1^1-371^ and FBP1^372-644^, by conducting an RNA-protein pull-down assay. RNA probes corresponding to positions nt 1–745 in the 5′ UTR of EV71 were biotinlyated and incubated with RD cell extracts expressing FLAG-fused FBP1, FBP1^1-371^ or FBP1^372-644^. The RNA-protein complex was pulled down with streptavidin beads and analyzed by immunoblotting. Results revealed that FLAG-FBP1 was pulled down by the beads, and a parallel experiment showed that FLAG-FBP1 was not pulled down by the beads if non-biotinylated EV71 5′ UTR RNA was used (Fig 6A, lane 2). A similar experiment also showed that FLAG-FBP1^1-371^, but not FLAG-FBP1^372-644^, bound to EV71 5′ UTR RNA and was pulled down by streptavidin beads (Fig 6A, lanes 4–9). We also used a biotinylated probe covering only the linker region (nt 636–745) in EV71 5′ UTR. We found that FLAG-FBP1 was pulled down by the beads (Fig 6B, lanes 1–3), confirming our earlier findings that FBP1 binds to the linker region in the EV71 5′ UTR [21]. Our results further showed that FLAG-FBP1^1-371^ binds to the 5′ UTR linker region probe (Fig 6B, lanes 4–6) but not FLAG-FBP1^372-644^ (Fig 6B, lanes 7–9), thus confirming that both FLAG-FBP1 and FLAG-FBP1^1-371^ can bind to the linker region in EV71 5′ UTR.

In order to assess whether FBP1^1-371^ competes with full-length FBP1 for binding to the linker region in the 5′ UTR, we conducted an *in vitro* competition binding assay, using both high and low amounts (to prevent saturation levels of proteins) of recombinant FBP1 and FBP1^1-371^ to define their respective binding kinetics. As can be seen in Fig 6C, the binding ability of FBP1 was not altered by increasing amounts of FBP1^1-371^, nor was FBP1^1-371^ affected by rising levels of FBP1. Similar results were also obtained when full-length EV71 5′ UTR RNA probe was utilized (S2 Fig). The results suggested that neither FBP1 nor FBP1^1-371^ competed with each other, and suggest that FBP1 and FBP1^1-371^ can bind to the linker region in the EV71 5′ UTR simultaneously. To assess whether FBP1 and FBP1^1-371^ associate with the EV71 5′ UTR simultaneously in infected cells, RNA immunoprecipitation and quantitative PCR (qPCR) were performed. RD cells were co-transfected with HA-FBP1/FLAG-FBP1^1-371^ or HA-vector/FLAG-FBP1^1-371^ for 48 hours, and subsequently infected with EV71 at a m.o.i. of 40. Cell lysates were prepared at 6 hours post-infection, and RNA-protein complexes were immunoprecipitated with anti-HA antibody. As shown in S3 Fig, FLAG-FBP1^1-371^ can be detected only in cells co-transfected with HA-FBP1/FLAG-FBP1^1-371^ (S3 Fig, lane 2), as compared to cells co-transfected with HA-vector/FLAG-FBP1^1-371^ (S3 Fig, lane 1). In addition, the interaction between HA-FBP1 and FLAG-FBP1^1-371^ was blocked when RNase A was added (S3 Fig, lane 6), which indicated that this interaction occurs in a RNA-dependent manner. In a parallel experiment, RD cell were co-transfected with FLAG-FBP1^1-371^/HA-FBP1 or FLAG-vector/HA-FBP1, and infected with EV71 at 48 hours post-transfection. Cell lysates were prepared at 6 hours post-infection, and RNA-protein complexes were immunoprecipitated with anti-FLAG antibody. HA-FBP1 was only detected in FLAG-FBP1^1-371^/HA-FBP1 overexpressing cells (S3 Fig, lane 10), but not FLAG-vector/HA-FBP1 overexpressing cells (S3 Fig, lane 9). The association between FLAG-FBP1^1-371^ and HA-FBP1 also disappeared when RNase A was applied (S3 Fig, lane 14). We further sought to ascertain whether EV71 5′ UTR existed in the precipitants, using qPCR. qPCR results revealed that immunoprecipitants from cells expressing HA-FBP1/FLAG-FBP1^1-371^ (S3 Fig, lane 2 and 10) co-precipitated with EV71 RNA. Taken together, these results demonstrate that FBP1 and FBP1^1-371^ associate with the EV71 5′ UTR simultaneously in infected cells.

We further conducted an enzymatic RNA footprinting assay to map the nucleotide sequences in the linker region that respectively interact with FBP1 and FBP1^1-371^. We used RNase T1, which specifically degrades single-stranded RNA at G residues, and RNase A, a pyrimidine nucleotide-specific endonuclease, to generate RNA footprinting patterns that can define the sequences protected by bound proteins. As shown in Fig 6D, FBP1 was able to protect the linker region RNA at nt 686–714 of EV71 (Fig 6D, lanes 2 and 5), while FBP1^1-371^ protected the linker region at nt 656–674 of EV71 (Fig 6D, lanes 3 and 6). The corresponding RNA sequences in the linker region that bind to FBP1 and FBP1^1-371^ are illustrated in Fig 6E. To further address whether FBP1 and FBP1^1-371^ can simultaneously bind to the EV71 5′ UTR linker region, gel mobility shift assays were conducted. Since FBP1 prefers to bind to AU-rich sequences, we introduced transversion mutations at the FBP1^1-371^ binding site (B1: nt 656–674 of EV71) or FBP1 binding site (B2: nt 686–714 of EV71) within the linker region probe (LR), thus converting these regions to GC-rich sequences (Fig 6F, top panel). Incubation of wild-type linker region probes (LR-B1B2) with FBP1^1-371^ or FBP1 can result in shifting of the probes (Fig 6F, lane 2 and 3), and supershifting of the probe was observed when FBP1^1-371^ and FBP1 were incubated simultaneously with the wild-type LR-B1B2 probe (Fig 6F, lane 4). In contrast, the mutations at the B1 or B2 binding sites (LR-mB1 and LR-mB2) strongly impaired the binding of FBP1^1-371^ or FBP1 to the probes; therefore, no shift in probes could be observed (Fig 6F, lane 6 and 11). When mutations were introduced at both the B1 and B2 binding sites (LR-mB1B2), no shifting or supershifting of the probes were detected after FBP1^1-371^ and FBP1 were added (Fig 6F, lane 14–16). Together, these results clearly demonstrate that FBP1 and FBP1^1-371^ can simultaneously bind to distinct sequences of the 5′ UTR linker region in a non-competitive fashion.

### FBP1^1-371^ acts as an additive ITAF to enhance viral IRES activity

**Fig 7:**
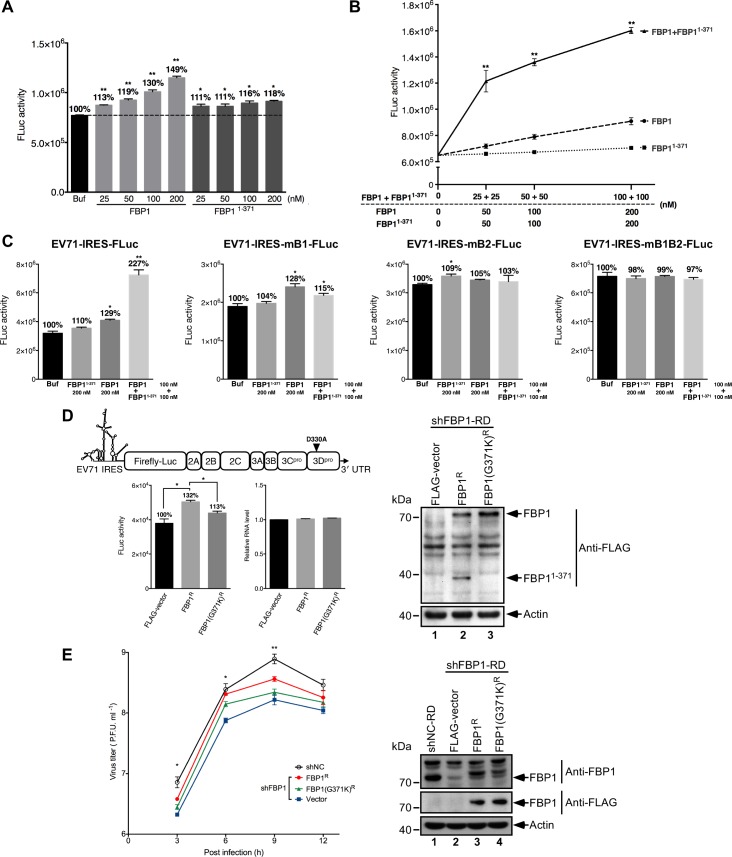
Additive effects of FBP1 and FBP1^1-371^ on IRES-driven translation. (A) FBP1 and FBP1^1-371^ impact on EV71 IRES activity *in vitro*. EV71 5′ UTR-FLuc RNA was translated with shFBP1-RD cytoplasmic extracts, in the presence of increasing amounts of recombinant FBP1 or FBP1^1-371^. Reactions without FBP1 or FBP1^1-371^ were used as controls. Luciferase activity exhibited by the reporter was monitored with a luminometer. (B) Additive effect of FBP1 and FBP1^1-371^ on EV71 IRES-driven translation *in vitro*. Recombinant FBP1, FBP1^1-371^ or a combination of FBP1 and FBP1^1-371^, were respectively added with EV71 5′ UTR-FLuc RNA to shFBP1-RD cytoplasmic extracts. A reaction without FBP1 or FBP1^1-371^ was used as a control. (C) Effects of FBP1 and FBP1^1-371^ binding to the linker region on EV71 IRES-driven translation *in vitro*. Recombinant FBP1, FBP1^1-371^ or a combination of FBP1 and FBP1^1-371^, were respectively added to wild-type EV71 5′ IRES-FLuc RNA, FBP1^1-371^ binding site mutant EV71-IRES-mB1-FLuc RNA, FBP1 binding site mutant EV71-IRES-mB2-FLuc RNA, or FBP1 and FBP1^1-371^ binding site-double mutant EV71-IRES-mB1B2-FLuc RNA in shFBP1-RD cytoplasmic extracts. A reaction without FBP1 or FBP1^1-371^ was used as a control. (D) EV71 replicon 3D^D330A^ (upper panel), which is defective in viral RNA replication, was used to determine the effects of FBP1 and FBP1(G371K) on viral protein translation. shFBP1-RD cells were transiently transfected with a vector control or plasmids expressing FLAG-tagged human FBP1 or FBP1(G371K) wobble mutant [FBP1^R^ and FBP1(G371K)^R^], which are resistant to the targeting of shFBP1. At 48 hours post-transfection, the cells were subsequently transfected with EV71 replicon 3D^D330A^ RNA. Luciferase activity exhibited by the cells were then monitored using a luminometer at 6 hours after the second transfection. Relative amounts of transfected replicon RNA and protein expression levels in each experimental set were also tested. The experiments conducted in A-D were repeated three times, and each sample was prepared in triplicate, while the results were analyzed statistically by Student’s t-test. Error bar: standard deviation; *: *p* <0.05 and **: *p* <0.01. (E) shFBP1-RD cells were transiently transfected with a vector control or plasmids expressing FLAG-tagged FBP1^R^ or FBP1(G371K)^R^. At 48 hours post-transfection, the cells were subsequently infected with EV71 at a m.o.i. of 40, and viral titers during the course of infection were titrated by plaque assays. Relative amounts of protein expression levels in each experimental set were also tested. The data were analyzed statistically by one-way ANOVA. Error bar: standard deviation; *: *p* <0.05 and **: *p* <0.01.

Given that FBP1 and FBP1^1-371^ bind to the EV71 5′ UTR linker region at different nucleotide positions in a non-competitive fashion, we sought to investigate whether FBP1^1-371^ exerted a comparable effect on EV71 IRES activity as full-length FBP1. Accordingly, an *in vitro* translation study using shFBP1-RD cytoplasmic extracts in the presence of recombinant FBP1 or FBP1^1-371^ was conducted. We found that adding recombinant FBP1 to cell extracts increased translation from an EV71 IRES reporter in a dose-dependent manner; at 200 nM, IRES activity increased by 49% over buffer control (Fig 7A). The addition of recombinant FBP1^1-371^ also appeared to increase IRES activity, but only by 11–18% over buffer control after 25–200 nM FBP1^1-371^ was added (Fig 7A). Upon EV71 infection, both cleaved and non-cleaved FBP1 were detected (Fig 1E), and therefore, we believed it would be interesting to evaluate the interactive effect of FBP1 and FBP1^1-371^ on EV71 IRES-mediated translation. When both FBP1 and FBP1^1-371^ were added to the reaction, luciferase activity results showed that IRES-driven translation was activated in a dose-dependent fashion, but more importantly, IRES activity was increased at levels substantially higher than those seen with FBP1 alone. In a reaction containing 100 nM each of FBP1 and FBP1^1-371^, IRES activity was found to increase by 80% over a reaction containing 200 nM of FBP1 (Fig 7B). These results demonstrate that in the presence of FBP1, FBP1^1-371^ can act as an additive component to enhance EV71 IRES activity. To further ascertain whether simultaneous binding of FBP1 and FBP1^1-371^ to the EV71 5′ UTR linker region is required for the additive effect on IRES translation, we introduced mutations at the FBP1 and FBP1^1-371^ binding sites within the linker region of EV71 5′ UTR-FLuc reporter RNA, similar to those described in Fig 6F, and subsequently tested the effects on IRES translation in shFBP1-RD cytoplasmic extracts in the presence of FBP1 alone, FBP1^1-371^ alone, or FBP1 + FBP1^1-371^. As shown in Fig 7C, the additive effect of FBP1 + FBP1^1-371^ on IRES translation was not seen with EV71-IRES-mB1-FLuc RNA, which contains transversion mutations in the FBP1^1-371^ binding site; EV71-IRES-mB2-FLuc RNA, which contains mutations in the FBP1 binding site; and EV71-IRES-mB1B2-FLuc RNA, which contains double mutations at both the FBP1 and FBP1^1-371^ binding sites. These results indicate that simultaneous binding of both FBP1 and FBP1^1-371^ to the EV71 5′ UTR linker region are required for additive enhancement of IRES translation.

To understand whether cleavage of FBP1 is essential for EV71 IRES-dependent translation, an EV71 replicon defective in RNA replication, 3D^D330A^, was used to ensure that observed luciferase activity is entirely dependent on IRES-driven translation. A shRNA-resistant FLAG-FBP1 (FBP1^R^) with wobble mutation was also generated to rescue FBP1 protein expression in shFBP1-RD cells. We found that IRES-mediated translation activity was enhanced by 32% over a FLAG-vector control by FBP1^R^ (Fig 7D), whereas FBP1(G371K)^R^, which contains a blocked viral 2A^pro^ cleavage site and is unable to generate FBP1^1-371^ (Fig 7D, lane 3), only displayed a moderate activating effect on EV71 IRES-driven translation (Fig 7D). Enhancement of IRES activity was not related to RNA replication activity of the EV71 replicon, and FBP1^R^ and FBP1(G371K)^R^ were observed to be expressed at comparable levels (Fig 7D, right panel). To further address whether the cleavage of FBP1 is important for EV71 virus yield in infected cells, shNC or shFBP1 RD cells that expressing vector control, FBP1^R^ or cleavage-resistant FBP1(G371)^R^ were infected with EV71 at a m.o.i. of 40, and the viral titers during the course of infection were measured by plaque assays. As shown in Fig 7E, the expression of FBP1^R^ can partially restore viral titers at 6 and 9 hours post-infection to levels comparable to shNC cells and shFBP1 cells that transfected with vector control. The expression of FBP1(G371K)^R^ can also rescue viral titers at 6 and 9 hours post-infection, but not to the same extent as FBP1^R^, and this indicates that cleavage of FBP1 and the additive effect from FBP1^1-371^ plays a key role in virus growth. The expression levels of endogenous FBP1, FBP1^R^ and FBP1(G371K)^R^ in each experiment were validated to be at comparable levels (Fig 7E, right panel). Together, these results show that FBP1^1-371^ works in tandem with FBP1 to increase IRES translation activity, and the existence of FBP1^1-371^ is essential for FBP1-mediated activation of EV71 IRES-driven translation, as well as virus replication.

## Discussion

**Fig 8:**
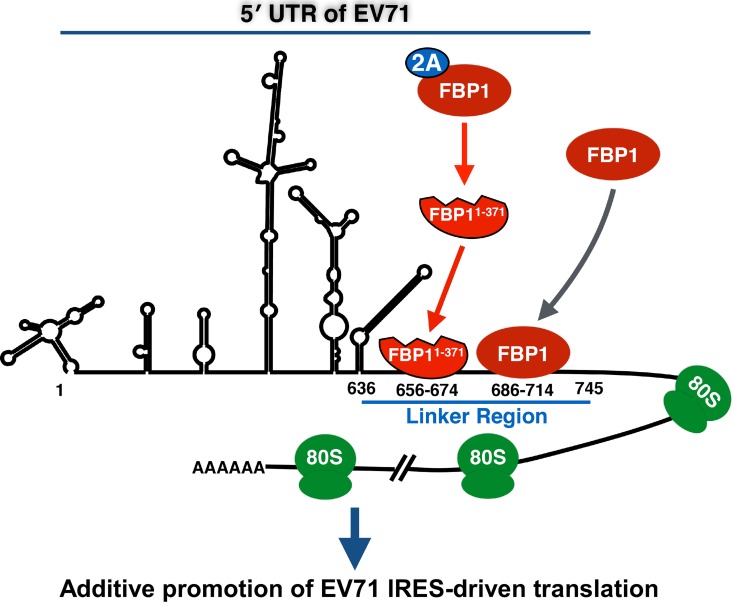
Proposed model for the functions of FBP1 and FBP^1-371^ in EV71 IRES-driven translation. During IRES-driven translation, FBP1 is recruited to the EV71 5′ UTR linker region at nt. 686–714 to enhance viral protein translation. As translation proceeds, the concentration of viral 2A^pro^ increases, leading to FBP1 cleavage and generation of a functional cleavage product, FBP1^1-371^, that can also serve as an ITAF in its own right. FBP1^1-371^ can also bind to the 5′ UTR linker region at a different site located at nt. 656–674, and acts additively with FBP1 to promote EV71 IRES-driven translation.

In this study, we identify a novel model in which EV71 not only recruits FBP1 to serve as an ITAF, but can also cleave FBP1 via the viral 2A^pro^ to generate a functional cleavage product, FBP1^1-371^, that acts additively with full-length FBP1 to enhance IRES-mediated translation (as summarized in Fig 8). These results provide important insights into viral recruitment and modulation of ITAFs during the infection process.

Due to the limited coding capacity of their genomes, picornaviruses typically utilize host factors to facilitate viral propagation. Most of these cellular factors originally reside in the nucleus, and picornavirus infection subsequently forces them to redistribute from the nucleus to the cytoplasm [24]. In order to achieve this, picornaviruses disrupt nuclear pore complexes (NPC) via cleavage of specific nuclear pore complex proteins (Nups) [48–52], such as Nup153, Nup98, and Nup62, and this disables key nuclear import and export pathways, thereby allowing the redistribution of nuclear-resident ITAFs [23], including nuclear factor FBP1 [21]. This recruitment of host proteins by EV71 and other picornaviruses is well-documented, although the viral cleavage of ITAFs such as Sam68 and Gemin5 have been shown to modulate the IRES activity of foot-and-mouth disease virus (FMDV), viral cleavage of ITAFs to yield functional cleavage products that can in turn cooperating with its full-length version to facilitate viral translation has not previously been reported. Here, we demonstrate that in RD cells, the cleavage of FBP1 during the course of EV71 infection is mainly carried out in the cytoplasm (Fig 1). To ascertain the mediator(s) of FBP1 cytoplasmic cleavage during EV71 infection, we first assessed known protein degradation processes induced by picornavirus infection. Unlike studies describing melanoma differentiation-associated protein 5 (MDA-5) and FBP2 cleavage upon picornavirus infection [41, 53], we showed that FBP1 cleavage was not processed by cellular mechanisms induced by viral infection, as treatment with proteasome inhibitor MG132 or the pan-caspase inhibitor QVD-OPh both failed to abolish cleavage, indicating that FBP1 cleavage is both a proteasome- and caspase-independent event (Fig 2). Previous studies have reported that a number of ITAFs, such as PTB, PCBP1, PCBP2, and AUF1, can be cleaved by poliovirus viral proteinase 3CD [30, 31, 54]. We therefore sought to ascertain if FBP1 was similarly cleaved by a viral proteinase. An *in vitro* cleavage assay confirmed that EV71 viral 2A^pro^ cleaves FBP1 at the Gly-371 residue to generate two cleavage products FBP1^1-371^ and FBP1^372-644^ (Figs 3 and 4). We overexpressed an uncleavable mutant, FBP1^G371K^, in EV71-infected RD cells, and observed that the mutant FBP1^G371K^ was resistant to viral cleavage by viral proteinase 2A (Fig 5). These results provide corroborating evidence that the Gly-371 residue is the authentic cleavage site. There is a remote possibility that Gly-371 may act as a required substrate element, but may not be the actual bond that is cleaved by viral proteinase 2A. To dispel concerns regarding this, we synthesized peptides from FBP1 aa 364–387 (WT: GQGNWNMGPPGGLQEFNFIVPTGK) and the G371K mutant (Mut: GQGNWNMKPPGGLQEFNFIVPTGK). Synthetic peptides were incubated with 5 μg of viral 2A^pro^ for 4 hours at 37°C, and the reactants were subsequently analyzed by LC-MS/MS (see S1 Methods). As shown in S4 Fig, the peptides, GQGNWNMGPPGGLQEFNFIVPTGK, GQGNWNM, and GPPGGLQEFNFIVPTGK, were detected after the WT peptide was subjected to viral 2A^pro^ treatment (S4A–S4C Fig). By contrast, only the GQGNWNMKPPGGLQEFNFIVPTGK peptide was detected after the Mut peptide was treated by viral 2A^pro^ (S4D Fig). These results convincingly demonstrate that Gly-371 is indeed the cleavage site of FBP1 for EV71 viral 2A^pro^.

In an earlier study, we observed the binding region of FBP1 to be at the linker region between nt 636 to 745 within the EV71 5′ UTR, and therefore an RNA-protein pull-down assay was conducted to test the binding capacity of FBP1 cleavage products to the EV71 5′ UTR. FBP1^1-371^ exhibited a pronounced binding affinity to the EV71 5′ UTR linker region as well (Fig 6A and 6B), and enzymatic footprinting and gel mobility shift assays further showed that FBP1^1-371^ and full-length FBP1 can simultaneously bind to this linker region at different binding sites without competition (Fig 6C, 6D and 6F). We also investigated whether the binding of FBP11-371 and FBP1 would outcompete other ITAFs, such as PTB, which promotes the initiation of picornavirus RNA translation via direct binding to stem loop V and its flanking regions in the poliovirus 5′ UTR [55]. The results in S5 Fig clearly demonstrated that neither FBP1 nor FBP1^1-371^ could outcompete PTB binding, even with increasing protein levels.

To understand how FBP1^1-371^ functions in mediating EV71 IRES activity, *in vitro* translation was conducted, and it was found that the joint presence of FBP1 and FBP1^1-371^ in the reaction greatly enhanced IRES activity in comparison to FBP1 alone (Fig 7A and 7B). This indicates that FBP1^1-371^ can act additively with full-length FBP1 to promote viral IRES-mediated translation. This additive promotion of EV71 IRES translation was abolished when mutations were introduced at the binding sites of FBP1 or FBP1^1-371^ within EV71 5′ UTR-FLuc reporter RNA (Fig 7C). This also indicates that the binding of FBP1 and FBP1^1-371^ proteins are required to achieve additive enhancement of IRES translation. In contrast to the type l IRES of EV71, the additive effect on IRES translation was not observed in type ll encephalomyocarditis virus (EMCV) IRES-driven translation, even when both recombinant FBP1 and FBP1^1-371^ were added to the reaction (S6 Fig).

Lower levels of viral protein translation and virus yield were observed in cells expressing the uncleavable mutant FBP1^G371K^, as compared to cells expressing wild-type FBP1, and this offers clear evidence that the additive effect of FBP1^1-371^ cannot be achieved by the expression of uncleavable FBP1 (Fig 7D and 7E). We note that the effects of FBP1, FBP1^1-371^ and FBP1^G371K^ on IRES-driven translation are relatively modest, and this may indicate that FBP1 is part of a group of ITAFs that interact with the EV71 IRES, and it is possible that these ITAFs may be able to compensate for the absence of FBP1 or the FBP1^1-371^ cleavage product.

Our data in Fig 6D and 6F and Fig 7 clearly demonstrate that the direct binding of FBP1 and FBP1^1-371^ to the EV71 5′ UTR promotes IRES-driven translation as well as viral yield. However, we cannot rule out the possibility that the cleavage of FBP1 may act as a switch to trigger IRES-dependent initiation of EV71 RNA synthesis. Whether the cleavage of FBP1 may confer advantages to promote IRES activity through this mechanism will be investigated in future research. Previously, PCBP2, an essential ITAF for viral protein translation, was reported to be cleaved by poliovirus viral proteinase 3CD, and this cleavage plays a role in mediating a switch in template usage from translation to RNA replication [31, 56]. Similarly, PTB, an ITAF that promotes the efficient initiation of poliovirus RNA translation, was shown to be cleaved by viral proteinase 3C, and this mediates a switch in template selection that favors viral translation over viral genome replication [30]. Another paradigm of ITAF cleavage has been described with the cellular mRNA decay protein AUF1, which can bind to stem-loop IV in the poliovirus 5′ UTR to negatively regulate viral propagation; however, this host antiviral response is partly inhibited through proteolytic cleavage of AUF1 by viral proteinase 3CD [20, 54]. We also demonstrated in previous research that FBP2, a negative ITAF, is cleaved in the late stage of EV71 infection through EV71-induced cellular mechanisms, including caspase activation, proteasome activity, and autophagy, to enhance viral IRES-mediated translation [41]. The cleavage of FBP1 differs from the paradigms mentioned above, as the effect of cleavage neither mediates viral genome template switching nor abolishes unfavorable factors for viral propagation; rather, the FBP1 cleavage event removes the KH4 and C-terminal domain of FBP1, but retains RNA binding ability to generate the positive ITAF FBP1^1-371^. Although the binding specificity of FBP1^1-371^ within the linker region differs from FBP1, and the cleavage product itself has a diminished positive effect on viral IRES-driven translation as compared to FBP1, we found that the joint presence of FBP1^1-371^ and full-length FBP1 contributed an additive enhancement of viral translation. We simulated the coexistence of FBP1 and FBP1^1-371^ during the middle stage of infection by adding recombinant versions of both proteins to an *in vitro* IRES activity assay, and found that FBP1^1-371^ can additively promote viral protein translation in a FBP1-dependent manner. To the best extent of our understanding, such an additive effect on viral IRES-mediated translation of a cleaved ITAF in tandem with its full-length progenitor has not been previously reported. However, further research will be needed to elucidate the detailed mechanisms by which FBP1^1-371^ exerts this additive effect.

In summary, our results show that EV71 viral proteinase 2A can cleave the positive ITAF, FBP1, to generate a cleavage product, FBP1^1-371^, that further acts additively with FBP1 to enhance viral IRES-driven translation as well as virus yield. These results point to a hitherto unknown role for ITAF cleavage, and provide important insight to the current understanding of viral recruitment and modulation of ITAFs.

## Materials and Methods

### Infection of RD cells and cell lysate preparation

Human embryonal rhabdomyosarcoma (RD) cells (ATCC, CCL-136) were maintained in Dulbecco’s modified Eagle medium (DMEM; Gibco, Grand Island, NY) containing 10% fetal bovine serum (FBS; Gibco) at 37°C. Cells were grown to 90% confluence and infected with EV71 strain Tainan/4643/98 at a multiplicity of infection (m.o.i.) of 40 in serum-free DMEM. Virus was allowed to adsorb at 37°C for 1 hour, after which cells were washed with phosphate-buffered saline (PBS) and incubated at 37°C in a medium containing 2% FBS. At specific time points post-infection, cells were washed with PBS and harvested to generate whole-cell lysates. Cell lysates were prepared as follows: cells were lysed with CA630 lysis buffer (150 mM NaCl, 1% CA630, 50 mM Tris-base [pH 8.0]) for 30 minutes on ice. Afterwards, lysates were centrifuged at 10,000 × *g* for 10 minutes at 4°C, and the supernatants were collected and stored at −80°C. Total protein concentrations were determined by the Bradford assay. For lysates used in pull-down assays, RD cells were transfected with designated FLAG-tagged FBP1 expression plasmids, and at 48 hours post-transfection, cells were washed with PBS and lysed with a 3-[(3-cholamidopropyl)-dimethylammonio]-1-propanesulfonate (CHAPS) lysis buffer (10 mM Tris-HCl [pH 7.4], 1 mM MgCl2, 1 mM EGTA, 0.5% CHAPS, 10% glycerol, 0.1 mM phenylmethylsulfonyl fluoride [PMSF], 5 mM β-mercaptoethanol) for 30 minutes on ice. Afterwards, cell lysates were centrifuged at 10,000 × *g* for 10 min at 4°C, and the supernatants were collected and stored at −80°C for further analysis.

### Plasmids and constructs

Plasmids pGL3-EV71 5′ UTR-FLuc and pCRII-TOPO-EV71 5′ UTR were previously constructed [21, 22]. Plasmid pGL3-EV71 5′ UTR-FLuc was subsequently adopted as a template for the construction of mutant pGL3-EV71 5′ UTR-FLuc plasmids, using a site-directed mutagenesis kit (Stratagene, La Jolla, CA) and the primers 5′-(CAGGGGCCGGCGGGCGGGCGGGCGTGGTTTTGTACCATTATCACTG)-3′ and 5′-(AACCACGCCCGCCCGCCCGCCGGCCCCTGTTGCACACCGGATGGCCA)-3′ for EV71-IRES-mB1-FLuc; primers 5′-(GTACGCGGCGGCGGCCCCGGGCGCCGGCGGGGGAAATTCATTTTGACCCTCAAC)-3′ and 5′-(GAATTTCCCCCGCCGGCGCCCGGGGCCGCCGCCGCGTACAAAACCAATAAATAGGTAAAC)-3′ for EV71-IRES-mB2-FLuc. Plasmid EV71-IRES-mB2-FLuc was then adopted as a template for the construction of EV71-IRES-mB1B2-FLuc plasmids, using a site-directed mutagenesis kit (Stratagene) and the primers 5′-(CGGTGTGCAACAGGGGCCGGCGGGCGGGCGGGCGTGGTTTTGTACGCGGC)-3′ and 5′-(GCCGCGTACAAAACCACGCCCGCCCGCCCGCCGGCCCCTGTTGCACACCG)-3′ Plasmid pFLAG-CMV2-FBP1 was generated as follows: FBP1 cDNA was amplified from plasmid pCMV-tag2B-FBP1 as described in [21], and the cDNA was then in-frame inserted into the pFLAG-CMV2 vector at the *Not*I and *Eco*RV sites. Plasmid pFLAG-CMV2-FBP1 was subsequently adopted as a template for the construction of mutant pFLAG-CMV2-FBP1 plasmids, using a site-directed mutagenesis kit (Stratagene) and the primers 5′-(AGTGTTCAGGCTAAAAATCCTAAAAAACCTAAACCTAAAAAACGAAAAAGAAAAAGAGGTCAAGGC)-3′ and 5′-(GCCTTGACCTCTTTTTCTTTTTCGTTTTTTAGGTTTAGGTTTTTTAGGATTTTTAGCCTGAACACT)-3′ for mutating glycine residues located between aa 345–362 to lysine; primers 5′-(GGAAGAGGTAGAAAACAAAAAAACTGGAACATGAAACCACCTAAAAAACTACAGGAATTTAAT)-3′ and 5′-(ATTAAATTCCTGTAGTTTTTTAGGTGGTTTCATGTTCCAGTTTTTTTGTTTTCTACCTCTTCC)-3′ for mutating glycine residues located between aa 364–380 to lysine; primers 5′-(AGAGGTAGAAAACAAGGCAAC)-3′ and 5′- (GTTGCCTTGTTTTCTACCTCT)-3′ for generating mutant G364K; primers 5′-(AGAGGTCAAAAAAACTGGAAC)-3′ and 5′-(GTTCCAGTTTTTTTGACCTCT)-3′ for generating mutant G366K; primers 5′-(TGGAACATGAAACCACCTGGT)-3′ and 5′-(ACCAGGTGGTTTCATGTTCCA)-3′ for generating mutant G371K; primers 5′-(GGACCACCTAAAGGACTACAG)-3′ and 5′-(CTGTAGTCCTTTAGGTGGTCC)-3′ for generating mutant G374K; and primers 5′-(CCACCTGGTAAACTACAGGAA)-3′ and 5′-(TTCCTGTAGTTTACCAGGTGG)-3′ for generating mutant G375K. For the construction of plasmids pFLAG-FBP1-HA and pFLAG-FBP1^G371K^-HA, FBP1 and FBP1^G371K^ cDNA with HA fused at the C-terminus were amplified with primers 5′-(AAGCTTGCGGCCGCGATGGCAGACTATTCAACA)-3′ and 5′-(GGTACCGATATCAGTTAAGCGTAATCTGGAACATCGTATGGGTAAGAGCCACCTTGGCCCTGAGGTGC)-3′ derived from pFLAG-CMV2-FBP1 and pFLAG-CMV2-FBP1^G371K^; the cDNAs were then in-frame inserted at the *Not*I and *Eco*RV sites of the pFLAG-CMV2 vector. Plasmids pFLAG-CMV2-FBP1 and pFLAG-CMV2-FBP1^G371K^ also served as templates for the construction of pFLAG-Hr-FBP1 and pFLAG-Hr-FBP1^G371K^ wobble mutants, using a site-directed mutagenesis kit (Stratagene) and the primers 5′-(CCATTCCTAGGTTCGCAGTCGGTATAGTTATAGGA)-3′ and 5′-(TCCTATAACTATACCGACTGCGAACCTAGGAATGG)-3′. For the construction of pFLAG-CMV2-FBP1^1-371^ and pFLAG-CMV2-FBP1^372-644^, FBP1 cDNA from pFLAG-CMV2-FBP1 was amplified using the primers 5′-(AAGCTTGCGGCCGCGATGGCAGACTATTCAACA)-3′ and 5′-(GGTACCGATATCAGTTATCCCATGTTCCAGTTGCC)-3′ for FBP1^1-371^, and primers 5′-(AAGCTTGCGGCCGCGATGCCACCTGGTGGACTACAG)-3′ and 5′-(GGTACCGATATCAGTTATTGGCCCTGAGGTGC)-3′ for FBP1^372-644^; the cDNAs were in-frame inserted between the *Not*I and *Eco*RV sites of the pFLAG-CMV2 vector. The plasmid for the EV71 replicon, 3D^D330A^, was generated from the EV71 replicon as previously described [41], using a site-directed mutagenesis kit (Stratagene) and the primers 5′-(AACATGGTGGCCTACGGGGATGCAGTGTTGGCTAGTTACCCCTTC)-3′ and 5′-(GAAGGGGTAACTAGCCAACACTGCATCCCCGTAGGCCACCATGTT)-3′.

### Expression and purification of recombinant proteins

Plasmids pET-23-EV71-3C (3C) and pET-23-EV71-m3C-C147S 3C (3C^C147S^) were constructed and purified as described previously [29]. Plasmid pGEX-6P-1-EV71-2A (2A) was constructed as follows. EV71 2A cDNA was amplified from the cDNA clone of EV71 and inserted into pGEX-6P-1 at the *EcoRI* and *Notl* sites. The primers used for 2A were 5′-(CCGGAATTCGGGAAATTTGGACAGCAG)-3′ and 5′-(CACGATGCGGCCGCTCCTGCTCCATGGCTTC)-3′. Plasmid pGEX-6P-1-EV71-2A served as a template for the construction of pGEX-6P-1-EV71-2AC110S (2A^C110S^), using a site-directed mutagenesis kit (Stratagene) and the primers 5′-(CCAGGGGATTCCGGTGGCATT)-3′ and 5′-(AATGCCACCGGAATCCCCTGG)-3′. pGEX-6P-1-EV71-2A (2A) and pGEX-6P-1-EV71-2AC110S (2A^C110S^) were purified using a GSTrap FF column (GE Healthcare, Waukesha, WI) according to the manufacturer’s instructions, and the GST-tag was removed with PreScission Protease (GE Healthcare). His-tagged recombinant FBP1 and FBP1^1-371^ were generated using a baculovirus expression system, and were purified as previously described [21]. Plasmid pBacPAK8-MTEGFP-His-FBP1 (FBP1) was previously constructed [21]. For the construction of pBacPAK8-MTEGFP-His-FBP1^1-371^ (FBP1^1-371^), cDNA was amplified by PCR using primers 5′-(GCTCTAGAATGGCAGACTATTCAACAGTGCCT)-3′ and 5′-(CGGGGTACCTCCCATGTTCCAGTTGCCTTG)-3′, and the PCR products were inserted into the pBacPAK8-MTEGFP-His vector (kindly provided by Dr. Tsu-An Hsu, National Health Research Institute, MiaoLi, Taiwan) at the *Xba*I and *Kpn*I sites.

### Coupled transcription/translation of [^35^S] methionine-labeled proteins

To produce [^35^S] methionine-labeled proteins, DNA fragments containing the T7 promoter and designated genes were amplified by PCR, and the designated proteins were produced with the TNT-coupled reticulocyte lysate system (Promega, Madison, WI) according to the manufacturer’s instructions.

### *In vitro* proteinase cleavage assay

RD cell extracts were prepared by washing cells with PBS and treating with CA630 lysis buffer for 30 minutes on ice without protease inhibitors, after which cells were harvested, centrifuged at 10,000 × *g* for 10 minutes at 4°C, and the supernatants collected. 30 μg of RD cell extract was incubated with 10 μg of recombinant viral proteinase (2A, 2A^C110S^, 3C and 3C^C147S^) in cleavage buffer (50 mM Tris-HCl, 50 mM NaCl, 5 mM DTT, 1 mM EDTA, pH 7.5) at a total volume of 15 μl at 37°C for 4 hours. The reactants were analyzed by immunoblotting for signals of proteolytic cleavage. To cleave the [^35^S]-labeled substrates, 5 μl of labeled protein from one TNT assay reaction was incubated with 10 μg of 2A proteinase in cleavage buffer with a total volume of 15 μl at 37°C for 4 hours. The reactants were analyzed by sodium dodecyl sulfate-polyacrylamide gel electrophoresis (SDS-PAGE) and autoradiography.

### Immunoblot analysis

Protein samples were resolved in SDS-PAGE gels, and proteins were subsequently transferred to polyvinylidene difluoride (PVDF) membranes (GE Healthcare). Membranes were blocked with Tris-buffered saline and 0.1% (vol/vol) Tween 20 containing 5% non-fat dry milk, and then probed with the indicated antibodies. Antibodies against FBP1 (611286 from BD Biosciences, Franklin Lakes, NJ; and GTX115154 from GeneTex, San Antonio, TX), eIF4G (GTX115154 from GeneTex), CstF-64 (sc-28201 from Santa Cruz Biotechnology, Santa Cruz, CA), FLAG (F3165 from Sigma, St Louis, MO), His (OB-05 from Calbiochem, LaJolla, CA), HA (H9658 from Sigma), PARP (sc-7150 from Santa Cruz), Lamin A/C (sc-20681 from Santa Cruz), GAPDH (H00002597-M01 from Abnova, Taiwan), and actin (MAB1502 from Millipore, Billerica, MA) were used. EV71 viral proteinase 3C and 3D monoclonal antibodies were generated from recombinant 3C^pro^ and 3D^pol^ proteins in our lab. For secondary staining, membranes were washed and incubated with HRP-conjugated anti-mouse antibody or HRP-conjugated anti-rabbit antibody. HRP was detected using the Western Lightning Chemiluminescence Kit (PerkinElmer Life Sciences, Boston, MA).

### *In vitro* transcription

Templates used for *in vitro* transcription were derived as follows: T7-EV71 5′ UTR DNA was excised from pCRII-TOPO-EV71 5′ UTR, using *Eco*RI restriction enzyme. T7-EV71 5′ UTR linker region RNA was derived by using the primers 5′-(TAATACGACTCACTATAGGGCCATCCGGTGTGCAACAGGGCAAT)-3′ and 5′-(GTTTGATTGTGTTGAGGGTCA)-3′ to amplify a DNA fragment containing the T7 promoter and the EV71 5′ UTR linker region sequence from pCRII-TOPO-EV71 5′ UTR. To generate EV71 5′ UTR-FLuc reporter RNA, plasmid pGL3-EV71 5′ UTR-FLuc was linearized using the *Xho*I restriction enzyme. To generate EV71 replicon 3D^D330A^ RNA, the EV71 replicon 3D^D330A^ plasmid was linearized using *Sal*I restriction enzyme. RNA transcript probes were synthesized using a MEGAscript T7 kit (ThermoFisher Scientific, San Jose, CA), according to the protocol recommended by the manufacturer. Biotinylated EV71 5′ UTR RNA probes were synthesized in a 20 μl reaction by adding 1.25 μl of 10 mM biotin-16-UTP (Roche, Mannheim, Germany) to the transcription reaction. RNA transcripts were purified using an RNeasy Mini kit (Qiagen, Chatsworth, CA). To generate Cap-FLuc reporter RNA, the primers 5′-(TAATACGACTCACTATAGGGATGGAAGACGCCAAAAACATAAAG)-3′ and 5′- TTACACGGCGATCTTTCCGCC)-3′ were used to amplify a DNA fragment containing the T7 promoter and the firefly luciferase gene from the pGL3-Basic vector, and Cap-FLuc reporter RNA was synthesized in a 20 μl reaction with adjustment of m7G(5′)ppp(5)G to GTP to a 4:1 ratio.

### Pull-down assay for biotinylated EV71 5′ UTR RNA with streptavidin beads

For pull-down assays, 200 μg of RD cell extracts (or the designated amount of recombinant FBP1 and FBP1^1-371^) and 12.5 pmol of biotinylated EV71 5′ UTR RNA was added to RNA mobility buffer (5 mM HEPES [pH 7.1], 40 mM KCl, 0.1 mM EDTA, 2 mM MgCl_2_, 2 mM dithiothreitol [DTT], 1 U RNasin, and 0.25 mg/ml heparin) and mixed to a final volume of 100 μl. The reactants were incubated at 30°C for 15 minutes, and 400 μl of streptavidin MagneSphere paramagnetic particles (Promega) were subsequently added. The mixture was allowed to incubate for 10 minutes at room temperature to pull down biotinylated ribonucleoprotein complexes. Pulled-down complexes were washed 5 times with RNA mobility buffer containing no heparin, after which 25 μl of 2× SDS sample buffer was added to the streptavidin beads at 95°C, and incubated for 10 minutes in order to dissociate the proteins from RNA. Protein samples were further resolved by immunoblot analysis

### Enzymatic RNA footprinting

The 5' end-labeled EV71 5' UTR linker region was incubated at 4°C for 10 minutes with 2 μg of full-length FBP1 or 1.14 μg of FBP1^1-371^ in binding buffer [57] containing 1 μl of 0.02 μg/μl RNase A or 0.02 U/ml RNase T1. The reactions were terminated with 10 μl of inactivation buffer (Ambion, Austin, TX). The cleavage products were separated in 12% acrylamide/7M urea gels, after which the gels were dried and subjected to a phosphor image scan. Nucleotide positions were determined through comparison with the Decade marker (Ambion). Footprinting experiments were performed in triplicate, with similar results derived overall.

### *In vitro* translation assay

shFBP1-RD stable cells were grown to 90% confluence in DMEM. Cells were washed and scraped with PBS, and then pelleted by centrifugation at 300 × *g* for 10 minutes at 4°C. After discarding the supernatants, the cell pellets were resuspended in 1.5x pellet volume of hypotonic lysis buffer (10 mM HEPES-KOH, pH 7.6, 10 mM KOAc, 0.5 mM Mg(OAc)_2_, 2 mM DTT, and 1x protease inhibitor cocktail [Roche]), placed on ice for 30 minutes, and then homogenized with a 27-gauge 1/2-inch needle. Cell extracts were centrifuged at 10,000 × *g* for 20 minutes at 4°C, and the supernatants were recovered and stored at −80°C. *In vitro* IRES activity assays were performed in a final volume of 25 μl containing 0.25 μg EV71 5′ UTR-Luc reporter RNA, 60% volume of RD shFBP1 cell extracts, 10 mM creatine phosphate, 50 μg/ml creatine phosphokinase, 79 mM KOAc, 0.5 mM Mg(OAc)_2_, 2 mM DTT, 0.02 mM hemin, 0.5 mM spermidine, 20 mM HEPES-KOH (pH 7.6), 20 μM amino acid mixture (Promega), 0.4 mM ATP (Promega), and RNase inhibitor. The reactants were incubated at 30°C for 90 minutes, and firefly luciferase activity was measured using the luciferase assay system (Promega).

### Reagents

The proteasome inhibitor MG132 and the lysosome inhibitor NH_4_Cl were purchased from Sigma. Pan-caspase inhibitor QVD-OPh was purchased from MP Biomedicals (Santa Ana, CA).

### Lentiviral vector preparation

The lentivirus vector pLKO_TRC005, carrying short hairpin RNA (shRNA) targeting nt 847 to 871 of human FBP1 mRNA (5′-CCAAGATTTGCTGTTGGCATTGTAA-3′), as well as the scramble control (5′-AATTTGCGCCCGCTTACCCAGTT-3′), were constructed according to the instructions of the Taiwan National RNAi Core Facility, Academia Sinica. For lentivirus preparation, 293T cells were co-transfected with LKO_TRC005-shRNA and the helper plasmids pMD.G and pCMVΔR8.91, using X-tremeGENE transfection reagent (Roche). Culture supernatant containing viral particles was harvested, and RD cells were transduced with shFBP1 lentivirus for 24 hours, then subject to selection with puromycin (5 μg/ml).

### Gel mobility assays and preparation of labeled RNA probes

RNA probes for use in RNA gel mobility shift assays (EMSAs) were generated by runoff transcription using bacteriophage T7 RNA polymerase, then purified with an RNeasy minikit (Qiagen), and subsequently labeled at the 5’ ends using T4 polynucleotide kinase and [γ-^32^P]ATP. EMSA was carried out to determine the interaction between the EV71 linker region RNA and FBP1, with methods described previously [57]. Briefly, 2 μg of FBP1 and/or 1.14 μg of FBP1^1-371^ was incubated for 30 minutes at 25°C with designated ^32^P-labeled RNA probes (1 × 10^5^ cpm). The reaction was carried out in binding buffer (10 mM HEPES [pH 7.5], 150 mM KCl, 0.5 mM EGTA, 2 mM MgCl_2_, 1 mM dithiothreitol, 1 unit RNasin,10% glycerol), and the final volume of the reaction mixture was 10 μl. The binding of FBP1 or FBP1^1-371^ to the viral RNA sequence was recognized by a slower migration of the labeled RNA probes.

### RNP immunoprecipitation

RD cells were transfected with HA vector, HA-FBP1, FLAG vector, or FLAG-FBP1^1-371^ constructs. At 48 hours post-transfection, cells were infected with EV71 at a m.o.i. of 40 PFU per cell. Cell lysates were harvested at 6 hours post-infection. For FLAG immunoprecipitation, cells were lysed with lysis buffer (50 mM Tris-HCl, pH 7.4, with 150 mM NaCl, 1 mM EDTA, and 1% Triton-X-100) for 30 minutes at 4°C, centrifuged at 12,000 × *g* for 10 minutes, and then incubated with anti-FLAG M2 affinity gel (Sigma) for 16 hours at 4°C. The protein complex was washed five times with wash buffer (50 mM Tris-HCl, pH 7.4, with 150 mM NaCl). HA immunoprecipitation was carried out with an Anti-HA Immunoprecipitation Kit (Sigma), according to the manufacturer’s instructions. Cell lysates were collected with CelLytic M Cell Lysis Reagent, incubated for 15 minutes, centrifuged at 20,000 × *g* for 15 minutes at 4°C, and subsequently incubated with anti-HA-Agarose (Sigma) for 16 hours at 4°C. The immunoprecipitation complex was washed five times with 1× IP buffer (Sigma) and once more with PBS. Each immunoprecipitation complex from anti-FLAG M2 affinity gel or anti-HA-Agarose was pelleted by centrifugation, and resuspended in 400 μl proteinase K buffer (100 mM Tris-HCl, pH 7.4, 150 mM NaCl, 12.5 mM EDTA, and 1% SDS) with 100 μg proteinase K (Sigma) for 30 minutes at 37°C. The RNA from the supernatant was extracted by TRIzol LS Reagent (Invitrogen) according to the manufacturer’s instructions. ReverTra Ace (TOYOBO) was used to reverse transcribed RNA to cDNA, and the Roche LightCycler 480 System and KAPA SYBR FAST qPCR Master Mix (Kapa Biosystems, Wilmington, MA) were deployed for quantitative detection of EV71 5′ UTR and actin. A set of EV71 5′ UTR primers was designed (forward 5’-CCCTGAATGCGGCTAATC-3’; reverse 5’-ATTGTCACCATAAGCAGCCA-3’). Primers for the actin control were also prepared (forward 5’-GCTCGTCGTCGACAACGGCTC-3’; reverse 5’-CAAACATGATCTGGGTCATCTTCTC-3’).

### Statistical analysis

Statistical significance was determined by performing two-tailed Student’s t-test and one-way ANOVA using Prism 6 software (GraphPad Software, San Diego, CA).

## Supporting Information

S1 Fig *In vitro* cleavage of CstF-64 by EV71 viral proteinase 3C. 10 μg of wild-type 3C^pro^ (3C) or mutant 3C^pro^ (3C^C147S^) viral proteinase was added to RD cell lysates and incubated for 4 hours at 37°C. Cleavage of CstF-64 was analyzed by immunoblotting, using CstF-64 antibody purchased from GeneTex.
(TIF)

S2 Fig *In vitro* competition assay of FBP1^1-371^ and FBP1 binding to EV71 5′ UTR RNA. Designated amounts of recombinant FBP1^1-371^ and FBP1 were incubated with the EV71 5′ UTR RNA probe, and analyzed by immunoblotting with anti-His antibody.
(TIF)

S3 Fig FBP1 and FBP1^1-371^ associate with EV71 RNA in infected cells. EV71-infected RD cells transfected with the designated proteins (HA-FBP1, FLAG-FBP1^1-371^, or both) were prepared and analyzed by ribonucleoprotein immunoprecipitation (RIP), using either HA-IP or FLAG-IP. EV71 RNA was analyzed by quantitative PCR.
(TIF)

S4 Fig LC-MS/MS analysis of the 2A^pro^ cleavage site for FBP1. Peptides corresponding to aa 364–387 for FBP1 (WT: GQGNWNMGPPGGLQEFNFIVPTGK) and the FBP1^G371K^ mutant (Mut: GQGNWNMKPPGGLQEFNFIVPTGK) were synthesized and respectively incubated with 2A^pro^ for 4 hours at 37°C. Reactants were then analyzed by LC-MS/MS. (A-C) peptides was detected after the WT peptide treated with viral 2A^pro^ and (D) peptide was detected after the Mut peptide was treated by viral 2A^pro^.
(TIF)

S5 Fig *In vitro* competition assay to EV71 5′ UTR RNA between FBP1, FBP1^1-371^ and PTB. Designated amounts of recombinant FBP1^1-371^ or FBP1 were incubated with fixed amounts of recombinant PTB to assess if FBP1 can outcompete the interaction between PTB and the EV71 5′ UTR RNA probe. Reactions were analyzed by immunoblotting with anti-His antibody.
(TIF)

S6 Fig Effect of FBP1 and FBP1^1-371^ on EMCV IRES-driven translation *in vitro*. EMCV IRES-FLuc RNA was translated with shFBP1-RD cytoplasmic extract in the presence of recombinant FBP1 or FBP1^1-371.^ Reactions without FBP1 or FBP1^1-371^ were used as controls. Luciferase activity exhibited by the reporter was monitored with a luminometer. The experiments conducted were repeated two times, and each sample was prepared in triplicate.
(TIF)

S1 Methods Supplementary Methods. (DOCX)
