# Supplementary figures and images for: Additive Promotion of Viral Internal Ribosome Entry Site-Mediated Translation by Far Upstream Element-Binding Protein 1 and an Enterovirus 71-Induced Cleavage Product
Source: PLoS Pathog. 2016 Oct 25;12(10):e1005959. doi: 10.1371/journal.ppat.1005959 (PMC5079569; doi:10.1371/journal.ppat.1005959)

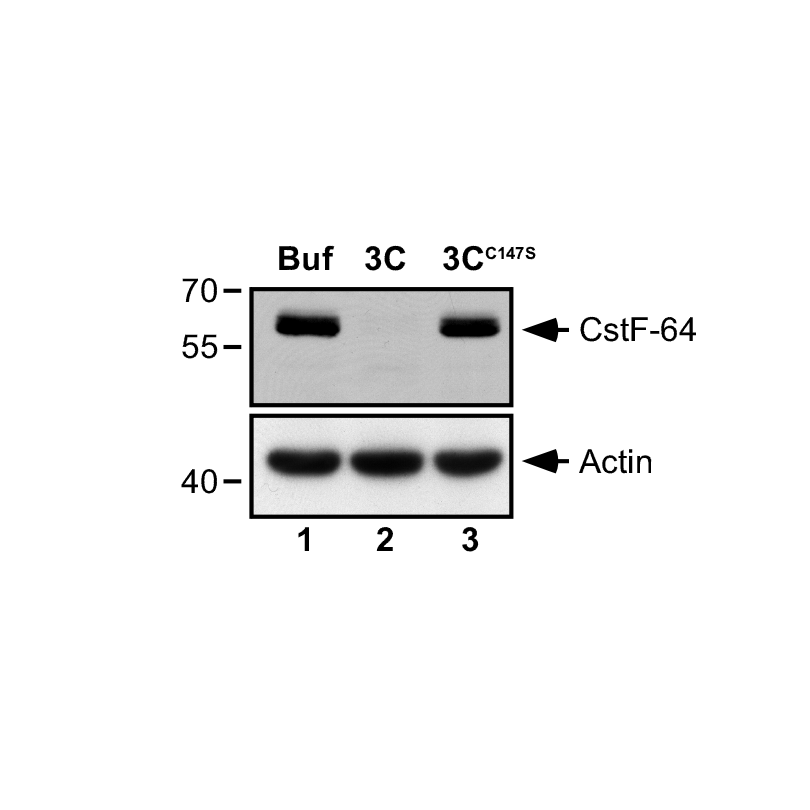

Supplement: S1 Fig — 10 μg of wild-type 3Cpro (3C) or mutant 3Cpro (3CC147S) viral proteinase was added to RD cell lysates and incubated for 4 hours at 37°C. Cleavage of CstF-64 was analyzed by immunoblotting, using CstF-64 antibody purchased from GeneTex. (TIF) [file ppat.1005959.s001.tif]

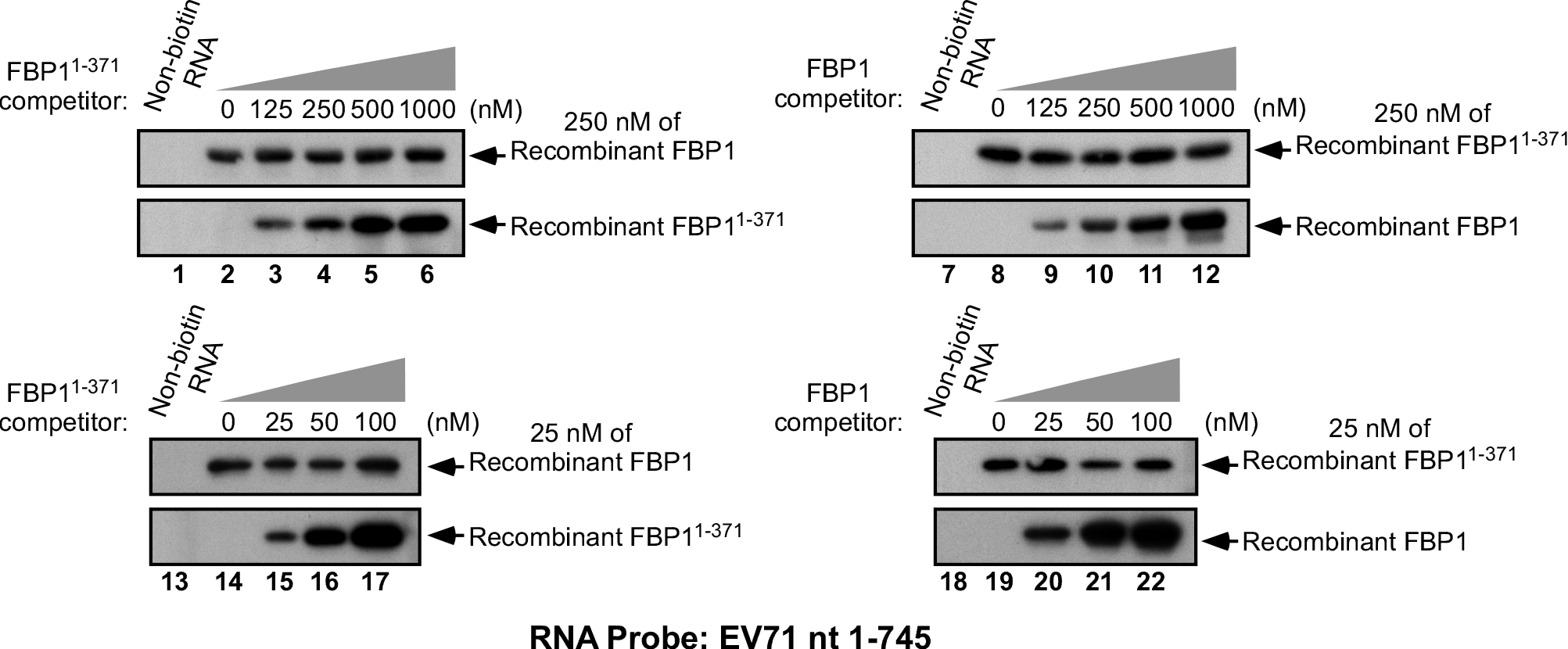

Supplement: S2 Fig — Designated amounts of recombinant FBP11-371 and FBP1 were incubated with the EV71 5′ UTR RNA probe, and analyzed by immunoblotting with anti-His antibody. (TIF) [file ppat.1005959.s002.tif]

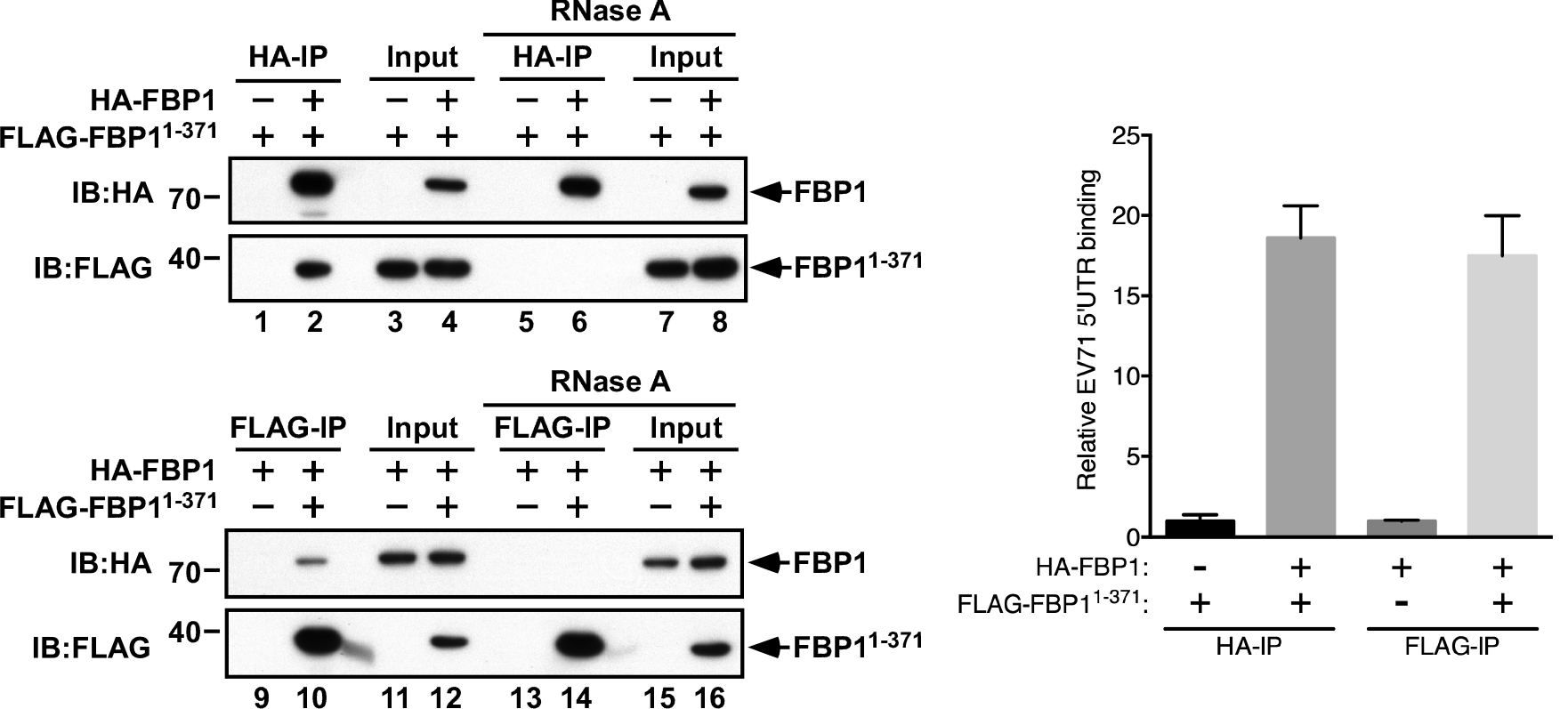

Supplement: S3 Fig — EV71-infected RD cells transfected with the designated proteins (HA-FBP1, FLAG-FBP11-371, or both) were prepared and analyzed by ribonucleoprotein immunoprecipitation (RIP), using either HA-IP or FLAG-IP. EV71 RNA was analyzed by quantitative PCR. (TIF) [file ppat.1005959.s003.tif]

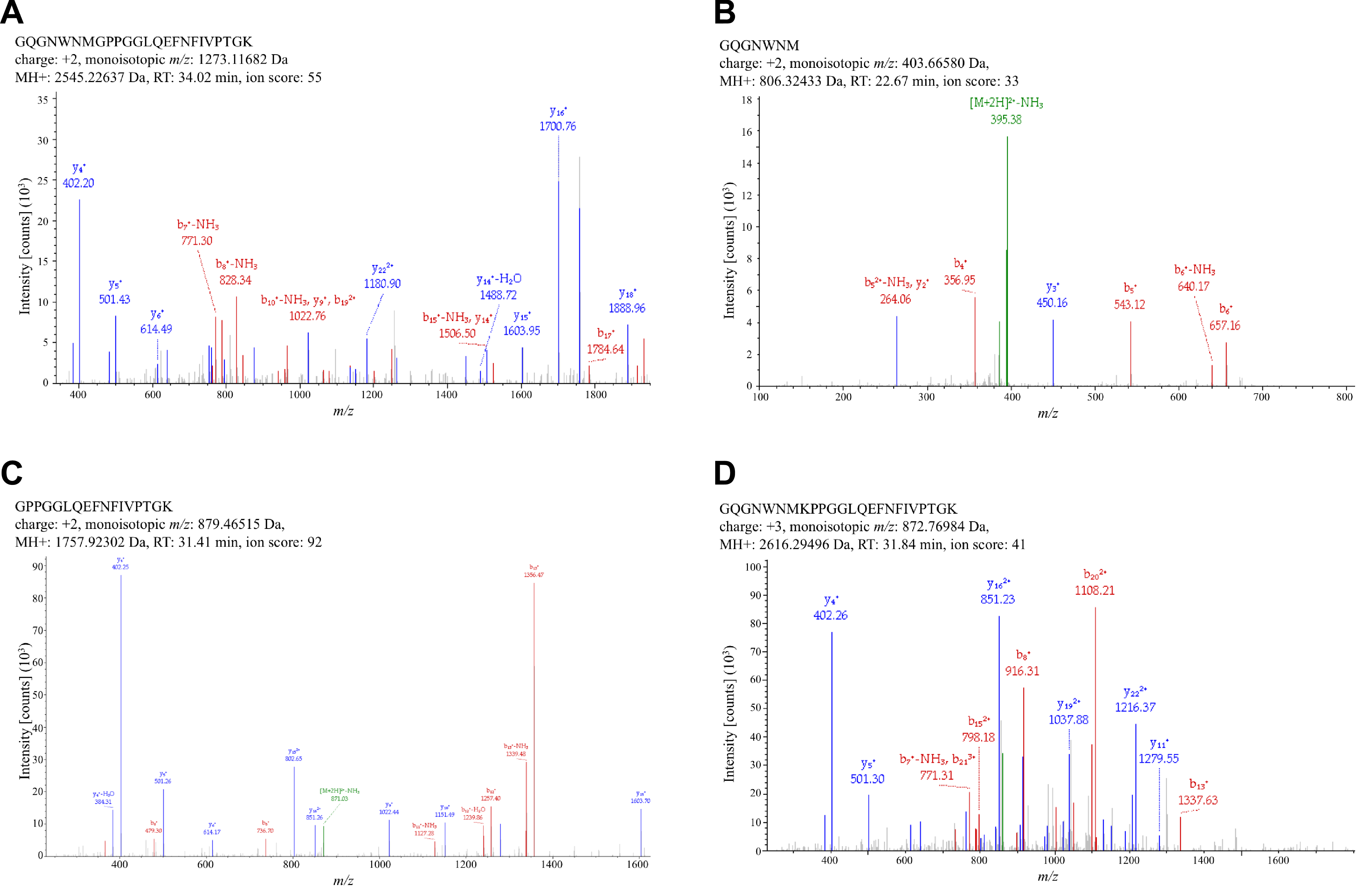

Supplement: S4 Fig — Peptides corresponding to aa 364–387 for FBP1 (WT: GQGNWNMGPPGGLQEFNFIVPTGK) and the FBP1G371K mutant (Mut: GQGNWNMKPPGGLQEFNFIVPTGK) were synthesized and respectively incubated with 2Apro for 4 hours at 37°C. Reactants were then analyzed by LC-MS/MS. (A-C) peptides was detected after the WT peptide treated with viral 2Apro and (D) peptide was detected after the Mut peptide was treated by viral 2Apro. (TIF) [file ppat.1005959.s004.tif]

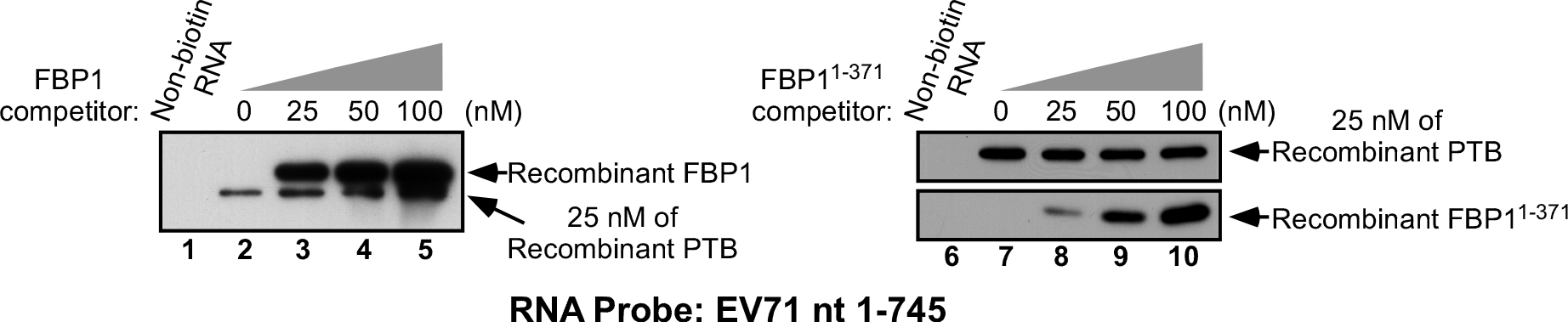

Supplement: S5 Fig — Designated amounts of recombinant FBP11-371 or FBP1 were incubated with fixed amounts of recombinant PTB to assess if FBP1 can outcompete the interaction between PTB and the EV71 5′ UTR RNA probe. Reactions were analyzed by immunoblotting with anti-His antibody. (TIF) [file ppat.1005959.s005.tif]

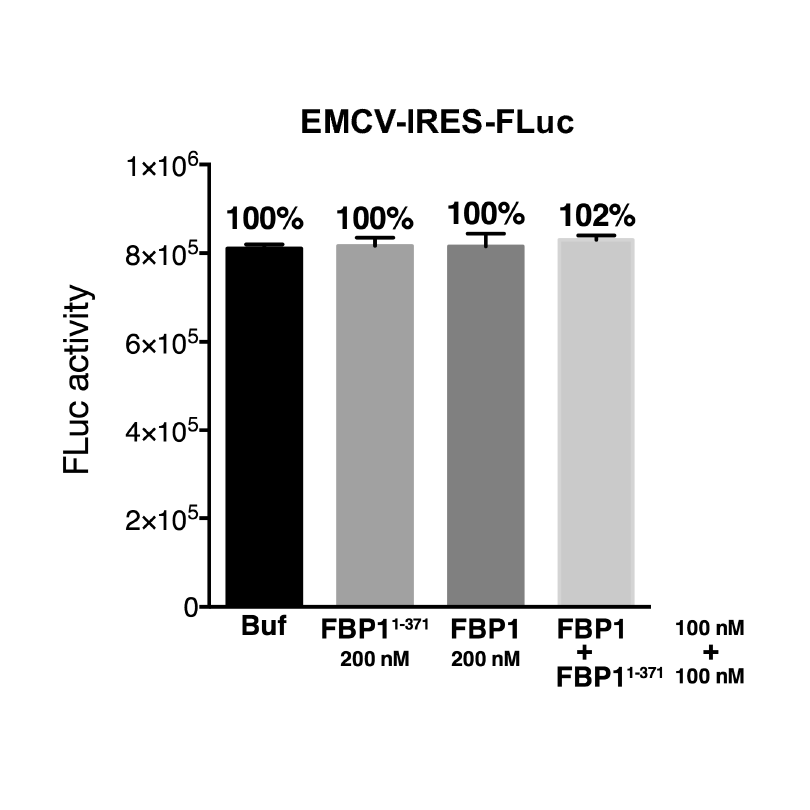

Supplement: S6 Fig — EMCV IRES-FLuc RNA was translated with shFBP1-RD cytoplasmic extract in the presence of recombinant FBP1 or FBP11-371. Reactions without FBP1 or FBP11-371 were used as controls. Luciferase activity exhibited by the reporter was monitored with a luminometer. The experiments conducted were repeated two times, and each sample was prepared in triplicate. (TIF) [file ppat.1005959.s006.tif]
