# Supplementary material for: Additive Promotion of Viral Internal Ribosome Entry Site-Mediated Translation by Far Upstream Element-Binding Protein 1 and an Enterovirus 71-Induced Cleavage Product
Source: PLoS Pathog. 2016 Oct 25;12(10):e1005959. doi: 10.1371/journal.ppat.1005959 (PMC5079569; doi:10.1371/journal.ppat.1005959)
Supplement: S1 Methods — (DOCX) [file ppat.1005959.s007.docx]

**Supplementary Methods**

**Reverse-phase liquid chromatography (RPLC) and mass spectrometry (MS)**

For peptide identification, each sample was reconstituted in buffer A (0.1% formic acid; Sigma-Aldrich, MO, USA), loaded onto a trap column (Zorbax 300SB-C_18_, 0.3 × 5 mm; Agilent Technologies, Taipei, Taiwan) at a flow rate of 0.2 μL/min in buffer A, and separated on a resolving 10-cm analytical C_18_ column (inner diameter, 75 μm) with a 15-μm tip (New Objective, MA, USA). Using a flow rate of 0.25 μL/min across the analytical column, the peptides were eluted using a linear gradient of 0-10% buffer B (99.9% ACN containing 0.1% formic acid) for 3 min, 10-30% buffer B for 35 min, 30-35% buffer B for 4 min, 35-50% buffer B for 1 min, 50-95% buffer B for 1 min, and 95% buffer B for 8 min. The RPLC apparatus was coupled online with a two-dimensional linear ion trap mass spectrometer (LTQ-Orbitrap Discovery, Thermo Fisher Scientific, NY, USA) managed using the Xcalibur 2.0 software package (Thermo Fisher Scientific). An electrospray voltage of 1.8 kV was applied. Intact peptides were detected by the Orbitrap at a resolution of 30000. The ion signal of (Si(CH_3_)_2_O)_6_H^+^ at *m/z* 445.120025 was used as an internal standard for mass lock. For MS analysis, we used a data-dependent acquisition mode that alternated between one MS scan and six MS/MS scans for the six most abundant precursor ions. For MS scans, the *m/z* scan range was set to 350-2000 Da. The *m/z* values selected for MS/MS scans were dynamically excluded for 3 min, and 5 × 10^4^ ions were accumulated and resolved in the ion trap to generate MS/MS spectra. Both MS and MS/MS spectra were acquired using one microscan with maximum fill times of 1000 ms and 100 ms for MS and MS/MS analyses, respectively. Automatic gain control was applied to prevent overfilling of the ion trap.

**Database searching for peptide identification**

For database searching, the obtained MS/MS spectra were analyzed using the Mascot algorithm (version 2.1, Matrix Science, MA, USA) against the database containing two peptides. One is derived from FBP1 amino acid 364-387 (GQGNWNMGPPGGLQEFNFIVPTGK) and the other one is the same peptide with substitution of Gly-371 for Lys residue (GQGNWNMKPPGGLQEFNFIVPTGK). The mass tolerances of the fragment and parent ions were set to 0.5 Da and 10 ppm, without digestion enzyme. No missed cleavage was permitted, and searches were performed with the parameters of variable oxidation on methionine (+15.99 Da). Peptides with Mascot ion score above 25 were considered to be identified.
